# NCCN guideline–concordant cancer care in sub-Saharan Africa: a population-based multicountry study of 5 cancers

**DOI:** 10.1093/jnci/djae221

**Published:** 2024-09-12

**Authors:** Nikolaus Christian Simon Mezger, Tobias Paul Seraphin, Robert Ballé, Mirko Griesel, Yvonne Walburga Joko-Fru, Lucia Hämmerl, Jana Feuchtner, Biying Liu, Annelle Zietsman, Bakarou Kamaté, Freddy Houéhanou Rodrigue Gnangnon, Franck Gnahatin, Dimitry Moudiongui Mboungou, Mathewos Assefa, Phoebe Mary Amulen, Gladys Chesumbai, Tatenda Chingonzoh, Cesaltina Feirreira Lorenzoni, Anne Korir, Pablo S Carvalho Santos, Jörg Michael Mezger, Haifa Kathrin Al-Ali, Rafael Mikolajczyk, Donald Max Parkin, Ahmedin Jemal, Eva Johanna Kantelhardt

**Affiliations:** Global and Planetary Health Working Group, Interdisciplinary Center for Health Sciences, Medical Faculty of the Martin Luther University Halle-Wittenberg, Halle, Germany; Global Public Health Department, Karolinska Institutet, Stockholm, Sweden; Global and Planetary Health Working Group, Interdisciplinary Center for Health Sciences, Medical Faculty of the Martin Luther University Halle-Wittenberg, Halle, Germany; Department of Gastroenterology, Hepatology and Infectious Diseases, University Hospital Duesseldorf, Medical Faculty at Heinrich-Heine-University, Duesseldorf, Germany; Global and Planetary Health Working Group, Interdisciplinary Center for Health Sciences, Medical Faculty of the Martin Luther University Halle-Wittenberg, Halle, Germany; Global and Planetary Health Working Group, Interdisciplinary Center for Health Sciences, Medical Faculty of the Martin Luther University Halle-Wittenberg, Halle, Germany; Global and Planetary Health Working Group, Interdisciplinary Center for Health Sciences, Medical Faculty of the Martin Luther University Halle-Wittenberg, Halle, Germany; African Cancer Registry Network, Oxford, UK; Global and Planetary Health Working Group, Interdisciplinary Center for Health Sciences, Medical Faculty of the Martin Luther University Halle-Wittenberg, Halle, Germany; Global and Planetary Health Working Group, Interdisciplinary Center for Health Sciences, Medical Faculty of the Martin Luther University Halle-Wittenberg, Halle, Germany; African Cancer Registry Network, Oxford, UK; African Cancer Registry Network, Oxford, UK; Dr AB May Cancer Care Centre, Windhoek Central Hospital, Windhoek, Namibia; African Cancer Registry Network, Oxford, UK; Registre des cancers de Bamako, Bamako, Mali; Service d’Anatomie Pathologique, CHU du Point G, Bamako, Mali; African Cancer Registry Network, Oxford, UK; Registre des Cancers de Cotonou, Cotonou, Benin; African Cancer Registry Network, Oxford, UK; Registre des Cancers d’Abidjan, Programme National de Lutte Contre le Cancer, Abidjan, Côte d’Ivoire; African Cancer Registry Network, Oxford, UK; Registre des Cancers de Brazzaville, Brazzaville, Republic of Congo; Global and Planetary Health Working Group, Interdisciplinary Center for Health Sciences, Medical Faculty of the Martin Luther University Halle-Wittenberg, Halle, Germany; African Cancer Registry Network, Oxford, UK; Addis Ababa City Cancer Registry, Radiotherapy Center, Addis-Ababa University, Addis Ababa, Ethiopia; African Cancer Registry Network, Oxford, UK; Kampala Cancer Registry, Department of Pathology, School of Biomedical Sciences, College of Health Sciences, Makerere University, Kampala, Uganda; African Cancer Registry Network, Oxford, UK; Eldoret Cancer Registry, Moi Teaching Hospital, Eldoret, Kenya; African Cancer Registry Network, Oxford, UK; Zimbabwe National Cancer Registry, Bulawayo, Zimbabwe; Radiotherapy Centre, Mpilo Central Hospital, Bulawayo, Zimbabwe; African Cancer Registry Network, Oxford, UK; Department of Pathology, Faculty of Medicine, Eduardo Mondlane University, Maputo Central Hospital, Maputo, Mozambique; African Cancer Registry Network, Oxford, UK; National Cancer Registry, Kenya Medical Research Institute, Nairobi, Kenya; Global and Planetary Health Working Group, Interdisciplinary Center for Health Sciences, Medical Faculty of the Martin Luther University Halle-Wittenberg, Halle, Germany; Department of Oncology and Hematology, ViDia Kliniken Karlsruhe, Karlsruhe, Germany; Krukenberg Cancer Center, University Hospital of Halle, Halle, Germany; Institute for Medical Epidemiology, Biometrics, and Informatics, Interdisciplinary Center for Health Sciences, Medical Faculty of the Martin Luther University Halle-Wittenberg, Halle, Germany; African Cancer Registry Network, Oxford, UK; International Agency for Research on Cancer, World Health Organization, Lyon, France; Surveillance and Health Equity Science, American Cancer Society, Atlanta, GA, USA; Global and Planetary Health Working Group, Interdisciplinary Center for Health Sciences, Medical Faculty of the Martin Luther University Halle-Wittenberg, Halle, Germany; Department of Gynaecology, Martin Luther University Halle-Wittenberg, Halle, Germany

## Abstract

**Background:**

To assess population-based quality of cancer care in sub-Saharan Africa and to identify specific gaps and joint opportunities, we assessed concordance of diagnostics and treatments with National Comprehensive Cancer Network Harmonized Guidelines for leading cancer types in 10 countries.

**Methods:**

Adult patients with female breast cancer, cervical cancer, colorectal cancer, non-Hodgkin lymphoma, and prostate cancer were randomly drawn from 11 population-based cancer registries. Guideline concordance of diagnostics and treatment was assessed using clinical records. In a subcohort of 906 patients with potentially curable cancer (stage I-III breast cancer, cervical cancer, colorectal cancer, prostate cancer, aggressive non-Hodgkin lymphoma [any stage]) and documentation for more than 1 month after diagnosis, we estimated factors associated with guideline-concordant treatment or minor deviations.

**Results:**

Diagnostic information based on guidelines was complete for 1030 (31.7%) of a total of 3246 patients included. In the subcohort with curable cancer, guideline-concordant treatment was documented in 374 (41.3%, corresponding to 11.7% of 3246 patients included in the population-based cohort): aggressive non-Hodgkin lymphoma (59.8%/9.1% population based), breast cancer (54.5%/19.0%), prostate cancer (39.0%/6.1%), colorectal cancer (33.9%/9.5%), and cervical cancer (27.8%/11.6%). Guideline-concordant treatment was most frequent in Namibia (73.1% of the curable cancer subcohort/32.8% population based) and lowest in Kampala, Uganda (13.5%/3.1%). Guideline-concordant treatment was negatively associated with poor ECOG-ACRIN performance status, locally advanced disease stage, origin from low Human Development Index countries, and a diagnosis of colorectal cancer or cervical cancer.

**Conclusions:**

The quality of diagnostic workup and treatment showed major deficits, with considerable disparities among countries and cancer types. Improved diagnostic services are necessary to increase the share of curable cancer in sub-Saharan Africa. Treatment components within National Comprehensive Cancer Network Guidelines for several cancers should be prioritized.

Cancer is among the three leading causes of premature death in most sub-Saharan African countries (1). To systematically improve oncological infrastructure and access to care, national cancer control plans have been rolled out. Indicators of the effectiveness of these plans, such as incidence, overall survival, and net survival, as collected by population-based cancer registries, are well defined (2) and could allow monitoring of the World Health Organization’s initiatives on cervical, childhood, and cancers (3-5). Data on the quality of clinical cancer care in the region, however—including diagnostics, treatment, and concordance with existing guidelines—had been published on hospital series but not at the population level. Therefore, we conducted a multicountry study on cancer care for five of the six most frequent malignancies in sub-Saharan Africa (6) in collaboration with registries under the umbrella of the African Cancer Registry Network (www.afcrn.org, supported by the Global Initiative for Cancer Registry Development) (7), the regional hub of the International Agency for Research on Cancer. Results on diagnostics, treatments, and outcomes for each of the 5 cancer types—female breast cancer, cervical cancer, colorectal cancer, non-Hodgkin lymphoma, and prostate cancer—have recently been published (8-13). This article provides a comprehensive overview of the uptake and quality of clinical cancer care across different cancer types and several countries in the region, most of which had little cancer-care infrastructure at the time (14). This work identifies major deficits in care and helps focus efforts within international collaborations. In light of restricted resources in sub-Saharan Africa, these comprehensive data on patient-level care enable us to prioritize action in addition to the World Health Organization initiatives.

## Methods

### Study sites

The 11 registries from Benin, Congo, Côte d’Ivoire, Ethiopia, Kenya, Mali, Mozambique, Namibia, Uganda, and Zimbabwe participating in this study cover a population of approximately 21 million inhabitants in capitol or large cities (except for Namibia’s, which is a nationwide registry). These registries record cancer cases from all oncological facilities, both public and private, in their respective areas (see Supplementary Figure 1, available online).

### Sampling of patients

During the study period (2012-2013), which was extended for individual registries back to 2010 and up to 2015 due to low patient numbers and for logistical reasons (see Supplementary Table 1, A and B, available online), 12 834 patients were registered with one of the above-named five cancer types (see Supplementary Table 2, available online). Cancer care was limited in sub-Saharan Africa during that time; therefore, we assumed stable conditions over those years (14). Regarding sample size calculations for individual cancer types, we considered a 2-sided 95% confidence interval (CI), with a width equal to 0.1 when the sample proportion of patients with adequate care was 0.500. We assumed a dropout rate of 33%; therefore, we aimed for 600 patients per cancer type as our random sample. We used the anonymous database from the 11 registries for the years 2012 and 2013, extending the study period for registries without enough cases. Ideally, we would have studied every case in the database, but we settled for samples of 60 cases at most sites because of financial and logistical limitations. For each cancer type, we applied a random number generated in Microsoft Excel to all cases. These numbers were then put into an order from smallest to largest. We entered the first 60 numbers as cases of the sample and checked the average age between the sample and that of all cases for the cancer concerned. As we observed no major differences in the age distributions, we assumed that the random sample represented the entirety of respective datasets. For registries that had a relatively small database, we employed all cases for the study.

As such, a random sample of 599 (non-Hodgkin lymphoma) to 892 (breast cancer) patients was drawn, resulting in a total cohort of 3784 patients (29.5% of 12 834 patients in the respective study periods and registries, see Supplementary Table 2, available online).

### Data-collection methods

**Figure 1.**
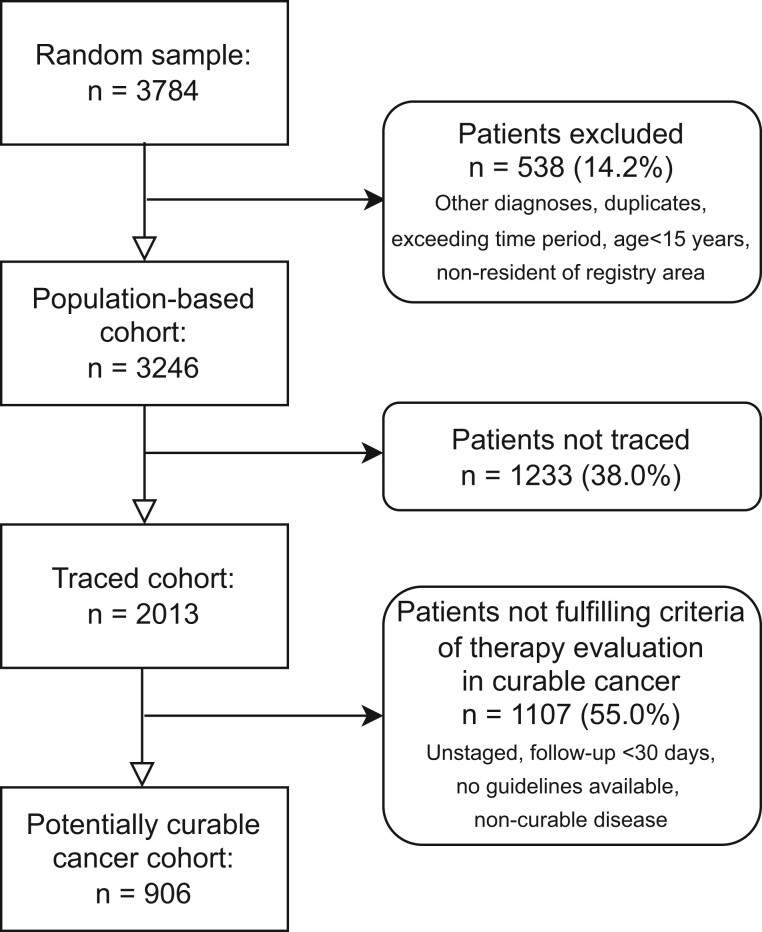
Flowchart of patients included in this study. Patients not traced: No information beyond cancer registry data available. For more information, refer to the detailed flowchart in Supplementary Figure 2 (available online).

The registry databases include basic demographic information and tumor characteristics but only minimal information about treatment. They often have incomplete follow-up data because there may be limited documentation on the case, usually created within a few months after pathological diagnosis. Currently, none of the registries in sub-Saharan Africa are equipped to collect disease stage, treatment information, or longitudinal data. For the random sample (N = 3784), additional information was obtained by searching clinical records and pathology reports, verifying or updating diagnosis, excluding any duplicates and false positives, and adding information about diagnostic procedures and treatment. Thus, 3246 patients were included as the population-based cohort (Figure 1). When possible, we contacted the patients or their relatives to ascertain treatment details in the case of in-country or international referral. The 2013 patients (62.0% of 3246) for whom a clinical record or pathology report was found through this active follow-up are subsequently referred to as “traced.” For the remaining 1233 patients not traced, no information beyond the basic registry data was available. During the data-collection process and since, cancer registries were supported logistically, financially, and through visiting experts.

### Data

As part of data collection, disease stage at diagnosis was recorded based on clinical and diagnostic information in the clinical records, according to internationally recognized classification group staging systems—namely, the Union for International Cancer Control staging system in breast cancer, colorectal cancer, and prostate cancer; the International Federation of Gynecology and Obstetrics (FIGO) staging in cervical cancer; and the Lugano/Ann Arbor and Binet staging systems in non-Hodgkin lymphoma (15-19). ECOG-ACRIN performance status at diagnosis and cancer type–specific data, such as hormone receptor status for breast cancer and prostate-specific antigen (PSA) tumor marker levels for prostate cancer, were collected. Select diagnostic parameters—namely, TNM classification in addition to hormone receptor status in breast cancer; FIGO staging in cervical cancer; TNM classification and lymph node assessment in stage I to III colorectal cancer; histopathological disease subtype and stage in non-Hodgkin lymphoma; and TNM classification, Gleason score, and PSA level as National Comprehensive Cancer Network (NCCN) prognostic factors in stage I to III prostate cancer—were evaluated as proxy indicators of diagnostic workup (17).

Further, seven aspects of cancer treatment were recorded in detail: surgery, external beam radiation therapy (RT), brachytherapy, chemotherapy, immunotherapy, hormone therapy, and targeted therapy. When specifics on procedures, such as type of hysterectomy or RT dose, were not further specified, but the clinical record reported “complete,” so we assumed that the treatment was performed in concordance with guidelines.

### Patient selection: potentially curable cancer

In this study, a main focus was evaluation of the quality of treatment in patients with potentially curable cancer, as defined by the NCCN Harmonized Guidelines for Sub-Saharan Africa (17,20-25). To this end, curable cancer includes stage I to III breast cancer, cervical cancer, colorectal cancer, and prostate cancer as well as aggressive non-Hodgkin lymphoma histopathological disease subtype, any stage. To identify patients with curable cancer, as a first step we regarded all 2013 traced patients among the 3246 patients in our population-based cohort. Among these traced patients, in breast cancer, cervical cancer, colorectal cancer, and prostate cancer, we excluded those patients with unknown disease stage (409/3246 [12.6%]) (see Supplementary Figure 2, available online). Patients with distant-stage disease (ie, stage IV breast cancer, cervical cancer, colorectal cancer, and prostate cancer) and generally noncurable, indolent non-Hodgkin lymphoma (eg, chronic lymphatic leukemia) were excluded (454/3246 [14.0%]). In traced non-Hodgkin lymphoma, we considered patients with known non-Hodgkin lymphoma subtypes, irrespective of disease stage, and excluded non-Hodgkin lymphoma subtypes for which harmonized guidelines are unavailable (eg, T-cell non-Hodgkin lymphoma) and unclassified non-Hodgkin lymphoma (75/3246 [2.3%]). In prostate cancer, we considered all patients with at least 1 of 3 NCCN prognostic factors: Gleason score, PSA level, and T category (17). To reduce survivor bias, we further excluded patients with follow-up of less than 30 days (169/3246 [5.2%]). Thus, of 3246 patients in our population-based cohort, 906 with curable cancer (27.9%) were eligible for evaluation of guideline-concordant treatment.

### Evaluation of guideline concordance in potentially curable cancer

For patients with curable cancer, we established a systematic evaluation scheme to assess concordance with guideline-recommended care. We used the NCCN Harmonized Guidelines for Sub-Saharan Africa as reference to assess therapy concordance to reasonable standards (17,20,25). Treatment was classified as guideline concordant, minor deviation, major deviation, or without curable potential (Table 1 [summary]). *Guideline concordance* was defined as NCCN’s harmonized guidelines’ “generally available standard of care.” *Minor deviation from guidelines* was defined, again according to NCCN, as “regional options that may be considered when availability precludes standard of care.” *Major deviation* was noted when a key therapy modality was missing. Each stage-dependent category includes key procedures and modalities required to reach a certain degree of guideline concordance with known survival benefit (for the detailed evaluation scheme, see Supplementary Table 3, available online). Non–guideline-concordant therapy was defined as “without curative potential.”

**Table 1.** Comparative therapy evaluation scheme for guideline concordance (17,21-25) of potentially curable cancer^a^

Potentially curable cancer	Guideline-concordant treatment and *minor deviation*	*Major deviation*	No cancer-directed therapy or therapy without curable potential	Therapy modalities required for cancer type
Breast cancer TNM classification I-III^b^	Multimodal approach, including surgery (with chemotherapy and/or external beam RT and/or hormone/targeted therapy)^b^	Surgery *without systemic approach* or systemic approach *without surgery*	No cancer-directed therapy identified	Surgery, chemotherapy, external beam RT, brachytherapy, hormone therapy, targeted therapy
Cervical cancer FIGO stage I-III^c^	(Radical) hysterectomy with or *without* pelvic lymphadenectomy or completed external beam RT plus completed brachytherapy with or *without* concurrent chemotherapy	Completed external beam RT *without brachytherapy*	Any other surgery; incomplete external beam RT or no cancer-directed therapy identified	Surgery, chemotherapy, external beam RT, brachytherapy
Colorectal cancer TNM classification I-III	Surgical removal plus >11 or *any* lymph nodes) (plus neoadjuvant or adjuvant RT and/or chemotherapy)	Surgery *without lymph nodes* (plus neoadjuvant or adjuvant RT and/or chemotherapy)	No surgery or colostomy/laparotomy only irrespective of adjuvant therapy or no therapy	Surgery, chemotherapy, external beam RT
Prostate cancer Known NCCN risk group and TNM classification I-III^c^	Radical prostatectomy or completed external beam RT Observation only or androgen-deprivation therapy accepted for low-risk-groups and lower life expectancy^d^	*Androgen-deprivation therapy* (only for high-risk-group)	Transurethral resection of the prostate or external beam RT (palliative dose) or chemotherapy only or no cancer-directed therapy	Surgery, external beam RT, hormone therapy
Diffuse, large B-cell lymphoma/Burkitt lymphoma Any stage or unknown	Diffuse, large B-cell lymphoma: CHOP with or *without* rituximab Burkitt: CODOX-M/IVAC with or *without* rituximab	*Other chemotherapy/immunotherapy regimen* with or without external beam RT	RT (except limited stage I) and/or surgery only or no cancer-directed therapy	Chemotherapy, immunotherapy (external beam RT)

a For details, see Supplementary Table 3 (available online). In column 2, minor deviations from guideline-concordant treatment are in italics and underlined; in column 3, major deviations from guideline-concordant treatment are in italic and underlined. CHOP = cyclophosphamide, doxorubicin, vincristine, and prednisolone; CODOX-M = cyclophosphamide, cytarabine, oncovin, doxorubicin, and methotrexate; FIGO = International Federation of Gynecology and Obstetrics; IVAC = ifosfamide, etoposide, and cytarabine; NCCN = National Comprehensive Cancer Network; RT = radiation therapy

b For breast cancer, the distinction between guideline concordance and minor deviation was not assessable because of lack of diagnostic parameters available (eg, lacking assessment of hormone receptor status, lacking imaging) and sophisticated requirements for therapy decision making (eg, incomplete information about axillary nodes and surgical margins) (24). Endocrine therapy was considered guideline concordant even for patients with unknown estrogen or progesterone receptor status (26).

c Although the NCCN Harmonized Guidelines for Sub-Saharan Africa propose intensive therapy approaches for FIGO stage IVA in cervical cancer and N1M0 in prostate cancer, these disease stages are considered at the verge of incurability and subject to individualized approaches that greatly stress the patient’s will and factors not present in our data (21). With respect to our retrospective evaluation and the limited resources available in sub-Saharan Africa, we categorized N1M0 cervical cancer as noncurable disease.

d Patient life expectancy was assessed using World Health Organization life tables from 2010 and 2015.

Showcasing nonconcordance compared between countries and cancers points to areas that need improvements. We included explanatory variables that highlight health system–related or patient-related barriers, such as Human Development Index (HDI) and age. Note that possible overtreatment was not the focus of the study. “Guideline-concordant” was the minimum therapy recommended.

### Statistical analysis

For statistical analysis, we used SPSS, version 28, statistical software (IBM Corp, Armonk, NY) and the RStudio, version 1.3.959, integrated development environment (Posit Software, PBC, Boston, MA). We implemented a multivariable modified Poisson distribution for binary data (also termed *Poisson regression with robust error variance*), as suggested by Zou (27), to assess the effect of select parameters (age continuous, early stage [TNM I/II, FIGO I-IIa, Ann Arbor I-II] vs locally advanced [TNM III and high-risk colorectal cancer TNM I/II, FIGO IIb-IIIb, Ann Arbor III/IV], low [0-1] vs advanced [2-4] grouped ECOG-ACRIN performance status, HIV status, cancer type, and low vs medium HDI) on guideline concordance of treatment. Guideline-concordant therapy and minor deviation from guideline-concordant treatment were grouped as *guideline-concordant treatment* for this analysis. The generalized linear regression was performed using the R package *geepack* (R Foundation for Statistical Computing, Vienna, Austria). Confidence intervals were derived from standard errors. The parameters used in the model were chosen by screening NCCN Harmonized Guidelines for Sub-Saharan Africa for joint prognostic factors of cancer types. A secondary regression was performed with the same parameters, except for registry instead of HDI. Relative risk (RR) and 95% CIs are reported. Statistical significance was set as α =  .05.

### Ethics

Ethical approval was granted by Martin Luther University Halle-Wittenberg (votum No. 2019-009) and the African Cancer Registry Network Review Committee (7.12.2017). The study was conducted in accordance with each registry’s regulations and was further approved by local ethical committees. The study protocol is in line with the Declaration of Helsinki.

### Role of the funding source

The study sponsors were not involved in the study design; in the collection, analysis, and interpretation of data; in writing the article; or in the decision to submit the paper for publication.

## Results

In the population-based cohort of 3246 patients, 2013 (62.0%) were traced. Tracing rates were highest in Cotonou, Benin (88.4%) and for cervical cancer (77.8%) and lowest in Brazzaville, Republic of Congo (27.0%) and for colorectal cancer (53.9%). Tracing rates also differed by sex, age group, and cancer type (see Supplementary Table 4, available online).

### Population-based cohort: baseline characteristics

Baseline characteristics and diagnostic information about the population-based cohort are displayed in Supplementary Table 5 (available online). Median follow-up was 201 days (interquartile range [IQR] =  16-658 days), ranging from 70 for non-Hodgkin leukemia (IQR = 0-370 days) to 330 days for breast cancer (IQR = 53-1134 days), and was less than 30 days for 447 patients. Disease stage at diagnosis was early (TNM I/II, FIGO I-IIa, Ann Arbor I-II) in 11.9% of patients, advanced (TNM III, high-risk colorectal cancer TNM I/II, FIGO IIb-IIIb, Ann Arbor III/IV) in 23.5% of patients, distant or metastasized (TNM/FIGO IV and low-grade non-Hodgkin lymphoma, any stage) in 14.0% of patients, missing in 12.6% of patients, and could not be identified in 38.0% of patients.

### Quality of diagnostics by NCCN guidelines in the population-based cohort

Disease stage for all cancer types and further cancer-specific indicators for quality of diagnostics, such as hormone receptor status in breast cancer, lymphoma subtype in non-Hodgkin lymphoma, and lymph node assessment in colorectal cancer, are depicted in Table 2. All selected prognostic factors recommended by the NCCN Harmonized Guidelines for Sub-Saharan Africa were available for 1030 of 3246 (31.7%) patients: 1) hormone receptor status and TNM stage were available in 15.2% of patients with breast cancer; 2) all 3 NCCN prognostic factors for prostate cancer—T category, PSA level, and Gleason score—as well as TNM stage I to III were known in 4.1% and TNM stage IV in 21.2% of patients with prostate cancer; 3) assessment of 12 or more lymph nodes and TNM stage I-III was documented in 1.8% and TNM stage IV identified in 14.3% of patients with colorectal cancer; 4) any FIGO stage was available in 67.6% of patients with cervical cancer; and 5) histopathological subtype of non-Hodgkin lymphoma was identified in 42.0% of patients. In contrast, diagnostics did not meet NCCN staging requirements in 509 of 3246 (15.7%) patients (ranging from 10.2% with unknown FIGO stage in cervical cancer to 23.8% in prostate cancer, with 1 or zero NCCN prognostic factors identified).

**Table 2.** Guideline concordance of prognostic factors relevant for treatment decisions in the population-based cohort (n = 3246), as recommended by the NCCN Harmonized Guidelines for Sub-Saharan Africa (17,20-25) and stratified by cancer type^a^

Diagnostics of prognostic factors	All, No. (%)	Breast, No. (%)	Cervical, No. (%)	Prostate, No. (%)	Colorectal, No. (%)	Non-Hodgkin lymphoma, No. (%)
	(n = 3246)	(n = 796)	(n = 630)	(n = 640)	(n = 673)	(n = 507)
Diagnostics of prognostic factors fulfilling NCCN staging requirements	1030 (31.7)	Estrogen receptor and/or progesterone receptor and/or ERBB2 status known TNM classification I-IV known 121 (15.2)	FIGO stage I-IV known 426 (67.6)	TNM classification I-III (NxM0/N0M0) known All 3 factors (T category, Gleason score, PSA level) assessed 26 (4.1) Known TNM stage IV (NxM1/N1Mx) 136 (21.2)	TNM classification I-III ≥12 lymph nodes assessed 12 (1.8) Known TNM classification IV 96 (14.3)	Subclassified non-Hodgkin lymphoma irrespective of Ann Arbor/Binet stage and irrespective of patient records traced 213 (42.0)
Diagnostics of prognostic factors partly fulfilling NCCN staging requirements	536 (16.5)	No estrogen receptor, progesterone receptor, or ERBB2 status known TNM classification I-IV known 275 (34.5)	Not applicable	2 of 3 factors assessed TNM classification I-III (NxM0/N0M0) known 51 (8.0)	1-11 lymph nodes or unknown number of lymph nodes assessed TNM classification I-III 129 (19.2)	Unclassified non-Hodgkin lymphoma Ann Arbor stage known 81 (16.0)
Diagnostics of prognostic factors not fulfilling NCCN staging requirements	509 (15.7)	TNM classification unknown irrespective of estrogen, progesterone, or hormone status 110 (13.8)	FIGO stage unknown 64 (10.2)	1 or no factors assessed TNM classification I-III (NxM0/N0M0) or TNM classification unknown 152 (23.8)	No lymph nodes examined TNM classification I-III or TNM classification unknown 126 (18.7)	Unclassified non-Hodgkin lymphoma Ann Arbor stage unknown 57 (11.2)
Medical records not traced	1171 (36.1)	Receptor status and TNM classification not assessable 290 (36.4)	FIGO stage not assessable 140 (22.2)	NCCN prognostic factors and TNM classification not assessable 275 (43)	Lymph node status and TNM classification not assessable 310 (46.1)	Unclassified Ann Arbor stage not assessable 156 (30.8)

a FIGO = International Federation of Gynecology and Obstetrics; NCCN = National Comprehensive Cancer Network; PSA = prostate-specific antigen.

### Treatment characteristics of the population-based cohort

**Figure 2.**
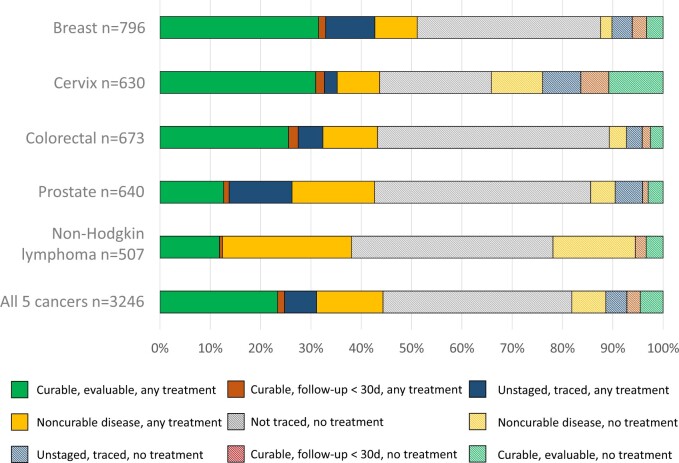
Treatment categories of patients adjusted for curability of disease, follow-up, staging and tracing in population-based cohort (n = 3246), stratified by cancer type. **Filled bars**: any cancer treatment documented; **striped bars**: no cancer treatment documented. Curable disease refers to stage I-III breast, cervix, colorectal and prostate cancer and aggressive Non-Hodgin lymphoma histopathological subtype, any stage. Noncurable disease refers to breast, cervix, colorectal and prostate cancer stage IV and indolent/unclassified Non-Hodgkin lymphoma. For details, see Supplementary Table 7 (available online). 30d = 30 days; CDT = cancer treatment; f-up = follow-up; NHL = Non-Hodgkin lymphoma.

In the population-based cohort, any cancer treatment was identified in 1446 of 3246 (44.5%) patients (ranging from 39.8% in non-Hodgkin lymphoma to 51.1% in breast cancer). Chemotherapy/immunotherapy was provided in 24.7% of cases, surgery in 24.2% of cases, RT in 13.4% of cases, and hormone therapy in 10.1% of cases (Supplementary Table 6, available online). Of 409 patients with unstaged disease, 207 (50.6%) received any cancer treatment; of 454 patients with noncurable disease, 318 (70.0%) were treated (Figure 2).

### Selection of patients with potentially curable cancer and baseline characteristics

A total of 906 patients with curable cancer, follow-up of at least 30 days, and NCCN Harmonized Guidelines for Sub-Saharan Africa available, were identified (see Figure 1; Supplementary Figure 2, available online): 277 patients with breast cancer (34.8% of 796 patients with breast cancer in the population-based cohort), 263 of 630 (41.2%) patients with cervical cancer, 100 of 640 (15.6%) patients with prostate cancer and at least 1 NCCN prognostic factor, 189 of 673 (28.1%) patients with colorectal cancer, and 77 patients with histopathologically highly malignant non-Hodgkin lymphoma subtype, disregarding stage (diffuse large B-cell lymphoma or Burkitt lymphoma, 15.2% of 507 patients) (see Table 3). An ECOG-ACRIN performance status of 2 or worse at diagnosis was documented in 16.6% of patients with curable cancer, HIV infection was documented in 14.2% of patients, and any imaging was identified in 44% of patients. Early stage (I or II) was identified in 29.4% of patients, stage III (and IV in non-Hodgkin lymphoma) in 67.4% of patients, and not identified in 3.2% of patients (non-Hodgkin lymphoma).

**Table 3.** Baseline characteristics and clinical diagnostics in patients with potentially curable cancer (n = 906), stratified by cancer type^a^

		All	Breast	Cervical	Prostate	Colorectal	Non-Hodgkin lymphoma
		N = 906	n = 277	n = 263	n = 100	n = 189	n = 77
Proportion of potentially curable cancer among population-based cohort, %		27.9	34.9	41.2	15.6	28.1	15.2
Population-based cancer registry, No. (%)	Abidjan, Côte d‘Ivoire	85 (9.4)	21 (7.6)	15 (5.7)	18 (18.0)	27 (14.3)	4 (5.2)
	Addis Ababa, Ethiopia	71 (7.8)	20 (7.2)	26 (9.9)	1 (1.0)	16 (8.5)	8 (10.4)
	Bamako, Mali	60 (6.6)	23 (8.3)	12 (4.6)	1 (1.0)	22 (11.6)	2 (2.6)
	Brazzaville, Congo	35 (3.9)	10 (3.6)	6 (2.3)	10 (10.0)	8 (4.2)	1 (1.3)
	Bulawayo, Zimbabwe	68 (7.5)	18 (6.5)	27 (10.3)	7 (7.0)	14 (7.4)	2 (2.6)
	Cotonou, Benin	84 (9.3)	44 (15.9)	11 (4.2)	18 (18.0)	11 (5.8)	—
	Eldoret, Kenya	104 (11.5)	31 (11.2)	46 (17.5)	10 (10.0)	17 (9.0)	—
	Kampala, Uganda	67 (7.4)	17 (6.1)	21 (8)	5 (5.0)	19 (10.1)	5 (6.5)
	Maputo, Mozambique	71 (7.8)	24 (8.7)	28 (10.6)	10 (10.0)	8 (4.2)	1 (1.3)
	Nairobi, Kenya	94 (10.4)	23 (8.3)	32 (12.2)	7 (7.0)	14 (7.4)	18 (23.4)
	Namibia (nation-wide)	167 (18.4)	46 (16.6)	39 (14.8)	13 (13.0)	33 (17.5)	36 (46.8)
HDI	Low	541 (59.7)	177 (63.9)	146 (55.5)	70 (70.0)	125 (66.1)	23 (29.9)
	Medium	365 (40.3)	100 (36.1)	117 (44.5)	30 (30.0)	64 (33.9)	54 (70.1)
Hospital type	Public	691 (76.3)	214 (77.3)	196 (74.5)	84 (84.0)	147 (77.8)	50 (64.9)
	Private	136 (15)	43 (15.5)	22 (8.4)	15 (15.0)	36 (19.0)	20 (26.0)
	Unknown	79 (8.7)	20 (7.2)	45 (17.1)	1 (1.0)	6 (3.2)	7 (9.1)
Follow-up, d	Median (IQR), mo	20 (7-43)	24 (9-45)	15 (7-36)	22 (7-46)	21 (9-42)	9 (4-41)
	≥30, <90	86 (9.5)	22 (7.9)	31 (11.8)	7 (7.0)	14 (7.4)	12 (15.6)
	≥90	820 (90.5)	255 (92.1)	232 (88.2)	93 (93.0)	175 (92.6)	65 (84.4)
Sex	Female	668 (73.7)	277 (100)	263 (100)	—	94 (49.7)	34 (44.2)
	Male	238 (26.3)	—	—	100 (100)	95 (50.3)	43 (55.8)
Age group, y	Median (IQR)	51 (40-62)	48 (40-57)	50 (40-59)	70 (62-77)	50 (39-61)	45 (36-55)
	15-29	58 (6.4)	15 (5.4)	13 (4.9)	—	20 (10.6)	10 (13)
	30-39	137 (15.1)	47 (17.0)	43 (16.3)	—	29 (15.3)	18 (23.4)
	40-49	219 (24.2)	88 (31.8)	72 (27.4)	1 (1.0)	41 (21.7)	17 (22.1)
	50-59	216 (23.8)	68 (24.5)	71 (27.0)	14 (14.0)	43 (22.8)	20 (26)
	60-69	144 (15.9)	37 (13.4)	39 (14.8)	30 (30.0)	35 (18.5)	3 (3.9)
	70-79	98 (10.8)	16 (5.8)	15 (5.7)	42 (42.0)	16 (8.5)	9 (11.7)
	≥80	34 (3.7)	6 (2.2)	10 (3.8)	13 (13.0)	5 (2.6)	—
Basis of diagnosis	Clinical	53 (5.8)	12 (4.3)	21 (8.0)	11 (11.0)	6 (3.2)	3 (3.9)
	Clinical, including ultrasonography, x-ray	53 (5.8)	28 (10.1)	11 (4.2)	6 (6.0)	5 (2.6)	3 (3.9)
	Biochemical	17 (1.9)	—	8 (3.0)	4 (4.0)	3 (1.6)	2 (2.6)
	Surgery	15 (1.7)	6 (2.2)	8 (3.0)	1 (1.0)	—	—
	Cytology or hematology	82 (9.1)	30 (10.8)	20 (7.6)	1 (1.0)	24 (12.7)	7 (9.1)
	Histology of primary site	659 (72.7)	200 (72.2)	180 (68.4)	75 (75.0)	143 (75.7)	61 (79.2)
	Unknown	27 (3)	1 (0.4)	15 (5.7)	2 (2.0)	8 (4.2)	1 (1.3)
ECOG-ACRIN performance status	0 or 1	283 (31.2)	97 (35.0)	85 (32.3)	29 (29.0)	52 (27.5)	20 (26.0)
	2-4	150 (16.6)	20 (7.2)	48 (18.3)	16 (16.0)	46 (24.3)	20 (26.0)
	Unknown	473 (52.2)	160 (57.8)	130 (49.4)	55 (55.0)	91 (48.1)	37 (48.1)
HIV status	Negative	155 (17.1)	41 (14.8)	50 (19)	16 (16.0)	34 (18.0)	14 (18.2)
	Positive	129 (14.2)	24 (8.7)	65 (24.7)	3 (3.0)	5 (2.6)	32 (41.6)
	Not identified	622 (68.7)	212 (76.5)	148 (56.3)	81 (81.0)	150 (79.4)	31 (40.3)
Stage	Early: TNM I/II, FIGO I-IIa, Ann Arbor I/II	266 (29.4)	111 (40.1)	72 (27.4)	24 (24.0)	43 (22.8)	16 (20.8)
	Advanced: TNM III (breast and prostate cancer), TNM I/II (high-risk colorectal cancer) and TNM III (colorectal cancer), FIGO IIb-IIIb, Ann Arbor III/IV	611 (67.4)	166 (59.9)	191 (72.6)	76 (76.0)	146 (77.2)	32 (41.6)
	Not staged	29 (3.2)	—	—	—	—	29 (37.7)
Imaging	Ultrasonography/x-ray	279 (30.8)	138 (49.8)	74 (28.1)	48 (48.0)	47 (24.9)	20 (26)
	Computed tomography/magnetic resonance imaging	120 (13.2)	11 (4.0)	29 (11.0)	19 (19.0)	65 (34.4)	15 (19.5)
	No imaging documented	507 (56)	128 (46.2)	160 (60.8)	33 (33.0)	77 (40.7)	42 (54.5)

a CRC = colorectal cancer; FIGO = International Federation of Gynecology and Obstetrics; HDI = Human Development Index.

### Treatment characteristics of patients with curable cancer

Among the subset of patients with curable cancer (n = 906), any cancer treatment was identified in 759 patients (83.8%), ranging from 74.1% in cervical cancer to 90.6% in breast cancer. Surgery was common (53.0% of 906 patients—specifically, 25.9% in cervical cancer, 76.2% in breast cancer, and 82% in colorectal cancer). Similarly, chemotherapy/immunotherapy was frequent, administered in half of the patients (50.7%—specifically, 65.8% in colorectal cancer, 66.8% in breast cancer, and 72.7% in non-Hodgkin lymphoma). One-third of patients received RT (29.6%—specifically, 19.6% in colorectal cancer, 31.4% in breast cancer, and 39.5% in cervical cancer). Finally, 35.7% of patients with breast cancer and 55% of patients with prostate cancer received any hormone therapy (see Supplementary Table 7, available online).

**Figure 3.**
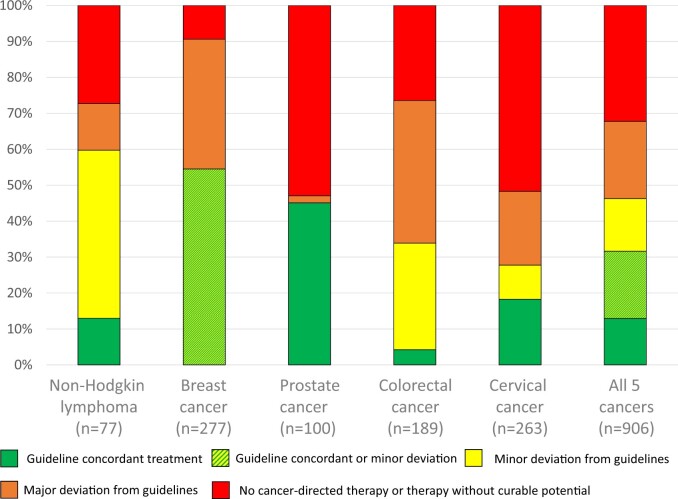
Guideline concordance (17,21-25) of treatment in patients with potentially curable cancer (n = 906), stratified by cancer type (for details, see Supplementary Table 6, available online). Evaluation of guideline concordance refers to Table 1 and Supplementary Table 3 (available online).

When applying the evaluation scheme (see Table 1 and Supplementary Table 3, available online), we found that 41.3% of the 906 patients with curable cancer received guideline-concordant treatment. The proportion of this treatment was highest in patients with non-Hodgkin lymphoma (59.8%) and breast cancer (54.5%) and lowest in patients with cervical cancer (27.8%). Patients with colorectal cancer had the highest proportion of major deviation from guidelines (39.7%); patients with prostate cancer had the second-highest deviation (34%). The highest proportion of patients without any therapy or therapy without any curable potential (lacking cancer treatment) was found in patients with cervical cancer (51.7%). Non-Hodgkin lymphoma, colorectal cancer, and prostate cancer had similar proportions of patients lacking cancer treatment (27.3%, 26.5%, and 27.0%, respectively), whereas in breast cancer, this proportion was as low as 9.4% (see Figure 3; Supplementary Table 5, available online).

### Population-based cancer registries

**Figure 4.**
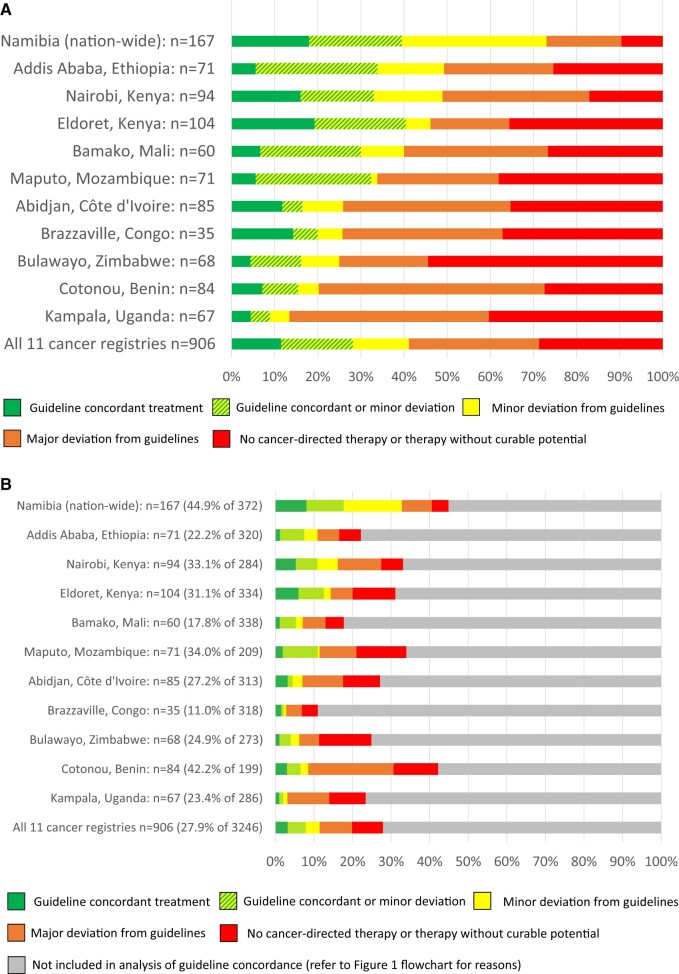
**A**) Guideline concordance (17,21-25) of treatment in patients with potentially curable cancer (n = 906), stratified by registry (for details, see Supplementary Table 6, available online). Evaluation of guideline concordance refers to Table 1 and Supplementary Table 3 (available online). **B**) Guideline concordance of therapy for patients with potentially curable cancer in the population-based cohort (n = 3246), stratified by registry. Percentages refer to proportion of patients evaluated for guideline-concordance among all patients. Evaluation of guideline concordance refers to Table 1 and Supplementary Table 3 (available online).

Across participating registries, the proportions of patients with staging information, curable (early/advanced) cancer, and sufficient follow-up for evaluation of guideline concordance in curable cancer varied markedly (44.9% of 372 patients in Namibia and 42.2% of 199 patients in Cotonou, Benin, vs 17.7% of 338 patients in Bamako, Mali, and 11.0% of 318 patients in Brazzaville, Republic of Congo) (Supplementary Figure 3, available online). At the population level, most patients received any cancer treatment in Nairobi, Kenya (66.5% of 284 patients); the fewest patients received any treatment in Bamako, Mali (34.6% of 338) and Brazzaville, Republic of Congo (17.0% of 318). Disparities in receipt by treatment modality were most pronounced in RT, with the fewest patients receiving any RT in Abidjan, Côte d’Ivoire; Bamako, Mali; Benin; Brazzaville, Republic of Congo; Bulawayo, Zimbabwe; and Maputo, Mozambique (2.5%-3.8% of 199-338 patients). The most patients receiving any RT were identified in Namibia (38.7% of 372) (for details, see Supplementary Table 8, available online). The separate analysis of receipt of guideline-concordant treatment in each country among the 906 patients with curable cancer showed that such treatment was most frequent in Namibia (73.1%) and Addis Ababa, Ethiopia (49.3%); it was lowest in Cotonou, Benin (20.2%) and Kampala, Uganda (13.5%) (Figure 4, A). At the population level, receipt of guideline-concordant treatment was most frequent in Namibia (32.8%) and least frequent in Kampala, Uganda (3.1%) (Figure 4, B). For details on treatment modalities, see Supplementary Table 9 (available online); for details on guideline-concordant treatment by cancer type and registry, see Supplementary Table 10 (available online).

### Factors associated with guideline-concordant treatment

**Figure 5.**
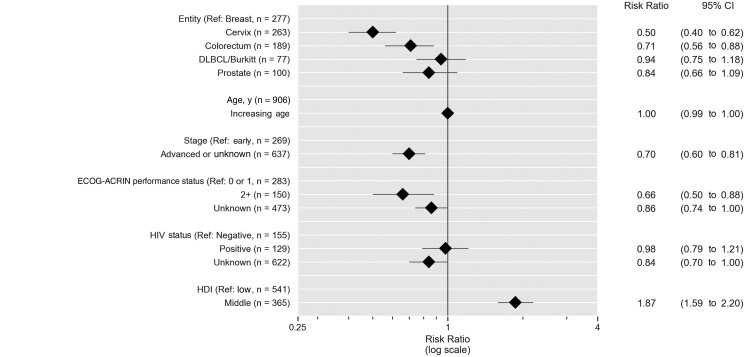
Factors associated with guideline-concordant treatment in patients with potentially curable cancer (n = 906, multivariable modified Poisson regression). *Guideline-concordant treatment* refers to combined guideline concordance or minor deviation from guidelines (17,21-25). Evaluation of guideline concordance refers to Table 1 and Supplementary Table 3 (available online). CI = confidence interval; DLBCL = diffuse large B-cell lymphoma.

Among the 906 patients with curable cancer, a poor ECOG-ACRIN performance status (≥2) and locally advanced or unknown (diffuse large B-cell lymphoma/Burkitt lymphoma) stage were inversely associated with guideline-concordant treatment (RR = 0.66, 95% CI = 0.50 to 0.88, and RR = 0.70, 95% CI = 0.60 to 0.81, respectively). Origin from a medium HDI country was found to be a strong predictor of guideline-concordant treatment (RR for middle-income HDI = 1.87, 95% CI = 1.59 to 2.20). Patients with colorectal cancer and cervical cancer were less likely than patients with breast cancer to receive guideline-concordant treatment (RR = 0.71, 95% CI = 0.56 to 0.88, and RR = 0.50, 95% CI =  0.40 to 0.62, respectively) (see Figure 5). We found no evidence for an association between age or positive HIV status and guideline-concordant treatment, but patients with unknown HIV status were found to be at higher risk of not receiving guideline-concordant treatment (RR = 0.84, 95% CI = 0.70 to 1.00). The secondary regression analysis, adjusted by registry, showed a similar ranking of relative risks for guideline-concordant treatment by registry (Figure 4, A; Supplementary Figure 4, available online).

## Discussion

### Summary of study

Our objective was to evaluate population-based, real-world quality of care among and across the most common cancers in ten countries using the NCCN Harmonized Guidelines for Sub-Saharan Africa. We present a synthesis of results previously presented separately for five cancer types to provide a real-life overview of the joint opportunities for improving diagnostics and treatment quality for sub-Saharan Africa. Through these comparisons, we aimed to derive common and specific suggestions for interventions to improve oncological care in sub-Saharan Africa. Targeting these findings can result in streamlined international cancer control efforts.

We traced records containing clinical information for two-thirds of the population-based cohort. Three in four patients had been diagnosed with stage III or IV cancer, but the actual proportion may be even higher given limited access to imaging, histopathological diagnostics, and poor documentation. Overall, seven in ten traced patients received any cancer treatment. Within the subcohort of patients with curable cancer, 41.3% received guideline-concordant treatment, which corresponds to 11.7% of the population-based cohort. The proportion of patients who received guideline-concordant treatment was higher in registries covering the populations of relatively wealthier countries. Our results describe the tenuous situation of patients with cancer in sub-Saharan Africa: Most are diagnosed with advanced-stage disease, and only a small proportion receive guideline-concordant treatment.

### Diagnosis

Our study showed that a high proportion of patients lack clinical staging, imaging, and histopathological analyses. These deficits can be explained by shortages of infrastructure, including immunohistochemistry services (28). Insufficient diagnostics may lead to understaging and inadequate treatment: the high share of missing Gleason score and T category information in prostate cancer and lack of histopathological subtype in non-Hodgkin lymphoma impede disease management. Only two in three patients with cervical cancer received appropriate clinical staging, and only one in three patients with breast or prostate cancer received the imaging necessary to assess disease extent. Regarding surgical staging for colorectal cancer, any lymph nodes were harvested and examined in one-third of patients, and a sufficient number of lymph nodes were harvested in a mere 12 patients. Nonsurgical staging in colorectal cancer was in part limited to ultrasonography and x-ray, with computed tomography and magnetic resonance imaging scans not available to all patients in the region.

The high share of metastatic disease in our study confirms other population-based findings from the region (29), which is particularly concerning in cancers for which cost-effective screening programs exist—namely, clinical breast examination and visual inspection of the cervix (30). A multicountry study on breast cancer from the region suggests that earlier detection and the availability of basic treatment could improve outcomes (33). Educational campaigns targeting benefits of early detection and treatment could be implemented (33).

### Treatment

The NCCN Harmonized Guidelines for Sub-Saharan Africa require a multidisciplinary approach that includes chemotherapy, immunotherapy and targeted therapy, surgery, external beam RT and brachytherapy, and hormone therapy (17,21-25). Many patients in our cohort received only fragmented care. Even more concerning, however, in more than 1 in 4 traced patients (and in virtually all nontraced patients), no cancer treatment was documented. In a worst-case but nonetheless probable scenario, these patients did not receive any cancer treatment.

Chemotherapy was a common therapeutic modality, presumably because it requires little infrastructure and is applied in all cancer types examined. Although the type and number of cycles of chemotherapy/immunotherapy was previously published by our group, our reports are limited with regard to dose intensity and the detailed analysis of substances used (9-11). Systemic therapy in sub-Saharan Africa is hampered by financial and logistical shortages, frequent stock unavailability, and lack of supportive drugs. As reported previously, CD20 antibodies, highly effective in B-cell lymphoma, were administered in just 20 patients with non-Hodgkin lymphoma (9). Most of these patients originated in Namibia. Also as reported previously, targeted therapy in breast cancer was even less prevalent, with 8 patients treated with trastuzumab (1.6% of traced patients, 22.8% of 35 patients with ERBB2-positive tumors) (11).

As further reported in our previous publications, surgery in both prostate cancer and cervical cancer was often inadequate—for example, transurethral resection of the prostate instead of radical prostatectomy in 58 patients with nonmetastatic prostate cancer and simple hysterectomy instead of radical hysterectomy with pelvic lymphadenectomy in 27 patients with FIGO IB-III cervical cancer (12,13). Many patients never received surgery. Initiatives to implement surgery for highly prevalent breast cancer at the primary and secondary health-care level, for example, might increase accessibility (32,34).

Functioning RT devices existed in just 4 of 10 participating countries during the study period: Ethiopia, Kenya, Namibia, and Uganda (35). In these countries, uptake of RT was as high as 38.7% (Namibia), as contrasted to uptake in countries without functioning facilities in a mere 2.5% to 3.8%. The long waiting time for RT in sub-Saharan Africa may lead to cancer progression (36). Within a mere 2 months, the proportion of advanced FIGO stages in cervical cancer increased markedly in an Ethiopian study, with a considerable number of patients dying while waiting (37). Both in curative and in palliative therapy, radiation is a necessary component in all 5 cancers included in our study, but only in 23 of 52 countries in sub-Saharan Africa any RT facilities were available in 2010 (35). Acknowledging this shortage, the NCCN provided revised therapy guidelines that apply in the absence of RT facilities (24).

Endocrine therapy is standard for hormone receptor–positive breast cancer, inexpensive, and has few side effects. Hormone receptor status was assessed for just 1 of 4 traced patients with breast cancer, whereas 1 in 3 patients received hormone therapy (11). This finding is unsatisfying because even in locally advanced breast cancer with unknown hormone receptor status, endocrine treatment has been recommended (38). In symptomatic, nonmetastatic prostate cancer, the NCCN Harmonized Guidelines for Sub-Saharan Africa propose a curative approach. Diverging from these recommendations, palliative androgen-deprivation therapy was the most frequently used approach in these patients (17). When more adequate cancer treatment is unavailable, suboptimal treatment, such as bilateral orchiectomy, may be cost-effective and beneficial (14).

### Quality of care

Among patients with curable cancer, the proportion receiving guideline-concordant treatment was highest in non-Hodgkin lymphoma and breast cancer, followed by prostate cancer, colorectal cancer, and cervical cancer. This finding reflects in part the more complex and expensive surgical requirements in prostate cancer, CRC, and cervical cancer compared with breast cancer (11-13). Obstacles related to systemic therapy impaired non-Hodgkin lymphoma and breast cancer care, and insufficient pathology services were a major hindrance to prostate cancer and non-Hodgkin lymphoma diagnosis. The absence of functioning radiation facilities primarily affected cervical cancer and, to a lesser degree, prostate cancer, breast cancer, and rectal cancer. All deficits varied by registry.

As we have published previously, patients with cervical cancer from countries with functioning radiation facilities received a higher quality of care, with important consequences: The hazard of dying was increased 9-fold in patients with untreated cervical cancer compared with patients who received guideline-concordant treatment (12). To a lesser extent, this pattern of survival benefit comparing guideline-concordant care with major deviations or no cancer treatment was found for the remaining 4 cancer types, as our group reported previously (8-13). In contrast, patients treated with minor deviations from guidelines showed only slightly impaired survival compared with strict guideline concordance. Across all cancer types, our observation that patients with early-stage disease and better clinical performance status were more likely to receive guideline-concordant treatment is compatible with allocation of resources to patients with a better prognosis. The lower proportion of patients receiving guideline-concordant treatment among patients not tested for HIV suggests that these patients might have had adverse access to medical services.

Our results in traced patients most probably overestimate the general quality of care. Many nontraced patients were found in registries associated with poorly financed health systems and are most likely underserviced or not serviced at all. Given the lack of any documentation beyond their initial cancer diagnosis, we assumed that the majority of these patients did not undergo any further procedures. In addition, we acknowledge that underprivileged groups, such as extremely poor individuals, are not covered because they never accessed the health system, and patients from rural areas are not represented. Thus, although just 1 in 9 patients in our cohort received guideline-concordant treatment, on national levels, we expect proportions to be even lower.

The participating registries cover a wide range of cultural, geographic, historical, and economic situations in sub-Saharan Africa. The different socioeconomic conditions and the resulting differences in medical resources available in the participating countries became quite apparent in access to diagnostics, shares of curable cancer, treatment modalities received, and overall share of guideline-concordant treatment. Living in a country with medium HDI increased the likelihood of receiving guideline-concordant treatment almost 2-fold. Similarly, the Cancer Survival in Countries in Transition group published differences in survival based on countries’ development level (39). A review published in 2018 showed disparities regarding the availability of clinical oncologists across sub-Saharan Africa. In Namibia, 1 oncologist was reported per 325 patients; in Ethiopia, 1 oncologist per 10 167 patients was reported (40). Corresponding to the much better economic situation than in most other countries, in Namibia, the health-care system offers cancer therapy such as chemotherapy and even immunotherapy as well as transportation free of charge (33). This finding corresponds to the high proportion of patients receiving guideline-concordant treatment in our study and may serve as a model for universal health coverage in oncology, as reflected by the best results of all registries examined.

NCCN guidelines were adapted for sub-Saharan Africa with the participation of African scientists. They showcase the minimum required standards for diagnostics and therapy. Our findings indicate that there are patients who receive such guideline-concordant care, even though the proportion remains low. Monitoring guideline-concordance treatment will enable to see changes over time, especially when new programs are initiated to improve cancer care. Monitoring can also identify areas where services are not easily accessible for patients.

### Limitations and strengths

We assume that cure is the most desirable and valuable goal of cancer therapy, so our in-depth analysis of guideline concordance is limited to patients with curable cancer. We did not, however, analyze in depth other cancer diagnoses that are considered not curable but for which low-cost, effective therapies with relatively few side effects are available in the region and recommended by NCCN—for example, stage IV prostate cancer and stage IV hormone receptor–positive breast cancer but also low-grade chronic lymphocytic leukemia.

The retrospective design of this study resulted in limitations. First, imprecise staging, poor documentation, and early loss to follow-up were frequent, similar to reports from other sub-Saharan African registries (2). Therefore, some findings are less precise than those from prospective studies. Due to incomplete documentation of clinical examinations and shortages in diagnostic workup, such as imaging in some cases (see Table 2), we may have underestimated the proportion of patients with early-stage and late-stage disease. Similarly, when the clinical documentation described treatment as complete but did not provide details, we accepted this classification as guideline-concordant. This decision may have led to misclassification of treatment and overestimation of guideline-concordant care in a few patients. It remains a subject of speculation whether patients not traceable (ie, no clinical records found) have been facing particularly inadequate care or left the registration area, (eg, to seek more appropriate treatment). Another reason for the high loss to follow-up is problematic archiving systems. Many study centers do not have well-established systems to document, trace, and archive cases, and they may lack electronic databases. Nevertheless, it seems more likely that no therapy and therefore no medical records were initiated for a large share of the patients for whom we did not find records. In patients with incomplete therapy, we presumed that a majority discontinued treatment. In this sense, we considered the high share of loss to follow-up and the constricted diagnostic and therapeutic data not only a limiting factor of this study but also a finding disclosing the concerning situation of cancer care in sub-Saharan Africa.

Our analysis may be limited due to residual confounding. Patients’ co-morbidities and socioeconomic factors, such as income and education, may have affected uptake of cancer therapy. Further, as is inherent to retrospective cohort studies, our study could not establish causation between reported factors and receipt of guideline-concordant treatment. Finally, the therapy evaluation scheme (Supplementary Table 2, available online) strictly followed the NCCN Harmonized Guidelines for Sub-Saharan Africa (33). We could not control, however, for all details regarding the technical realization of respective therapy modalities, such as timeliness of RT and dose-appropriate administration of systemic therapy, because of inconsistent documentation. Given the manifold hindrances to access to and uptake of cancer care discussed here, we considered the presented share of guideline-concordant, quality care an overestimate. In a companion study started 2024, our research group, in cooperation with the African Cancer Registry Network, intends to account for some of these limitations.

There are important strengths to our study, as well. First, we included a population-based random sample of patients from 11 registries involving public and private institutions, not just patients referred to specialists and patients with and without treatment. Second, the study involved a variety of countries in sub-Saharan Africa, reflecting a wide range of socioeconomic conditions and different health services in the region. This study created a link between the NCCN Harmonized Guidelines for Sub-Saharan Africa and therapy actually received for patients with the most common cancer types in real-world sub-Saharan Africa based on cross-sectional and longitudinal data.

Our data demonstrate the diverse and overall dissatisfying quality of clinical care in 11 oncological centers and in 5 different cancers at the population level. This study may serve as a baseline for targeting general diagnostic and therapeutic as well as site-specific and cancer-specific gaps. It seems sensible to highlight central components within the NCCN Harmonized Guidelines for Sub-Saharan Africa and synergistic investments, which should be prioritized in further development. In our opinion, these central components include strengthening pathological services (eg, hormone receptor testing in breast cancer, subclassification in non-Hodgkin lymphoma, and determining Gleason score in prostate cancer), ensuring availability of radiation, and training surgeons to perform appropriate procedures. In addition, patient-perceived enablers and barriers to care must be taken into account. Population-based cancer registries in sub-Saharan Africa should be adequately supported to monitor progress over time.

## Supplementary Material

djae221_Supplementary_Data

## Data Availability

Individual deidentified participant data may be shared upon request to investigators whose proposed use of the data has been approved by an independent review committee (“learned intermediary”) identified for this purpose.
