# Supplementary material for: NCCN guideline–concordant cancer care in sub-Saharan Africa: a population-based multicountry study of 5 cancers
Source: J Natl Cancer Inst. 2024 Sep 12;117(1):120–33. doi: 10.1093/jnci/djae221 (PMC11717413; doi:10.1093/jnci/djae221)
Supplement: djae221_Supplementary_Data [file djae221_supplementary_data.zip › djae221_Supplementary_Data/Supplement_revised_2208.pdf]

Supplementary Material

Supplementary material: Table of content

Supplementary Table 1A and 1B. 1A: Year of diagnosis and contributing population-based cancer registries. 1B: Year of diagnosis and specific cancer type.

Supplementary Table 2. Population-based cancer registries (PBCR) and study population characteristics.

Supplementary Figure 1. Map of Sub-Saharan Africa.

Supplementary Figure 2. Detailed flow chart.

Supplementary Table 3. Detailed comparative therapy evaluation scheme for guideline concordance of potentially curable cancer.

Supplementary Table 4. Traced patients in population-based cohort (n=3246). Stratified by age group, sex, cancer type and population-based cancer registry.

Supplementary Table 5. Baseline characteristics and clinical diagnostics in population-based cohort (n=3246), stratified by cancer type.

Supplementary Figure 3. Treatment categories of patients adjusted for curability of disease, follow-up, staging and tracing in population-based cohort (n=3246), stratified by cancer registry

Supplementary Table 6. Treatment by type in population-based cohort (n=3246), stratified by registry.

Supplementary Table 7. Treatment by type in therapy evaluation cohort (n=906), stratified by registry.

Supplementary Table 8. Details on overall treatment and guideline concordance in patients with potentially curable cancer (n=906), stratified by cancer type.

Supplementary Table 9. Overall treatment and guideline concordance in patients with potentially curable cancer (n=906), stratified by registry.

Supplementary Table 10. Proportion of guideline-concordant treatment (GCT) by cancer type and registry in patients with curable cancer (n=906).

Supplementary Figure 4. Factors associated with guideline concordant therapy treatment (GCT) in patients with potentially curable cancer (n=906, multivariable modified Poisson regression; adjusted by registry)

Supplementary Table 1A+1B. Year of diagnosis by registry and cancer type

Supplementary Table 1A. Year of diagnosis and contributing population-based cancer registries. Population-based cohort (n=3246).

		Patients per year of diagnosis						
		2010	2011	2012	2013	2014	2015	Total patients
Registry	Abidjan	2	8	99	204	0	0	313
	Addis	2	2	202	9	105	0	320
	Bamako	1	5	137	193	2	0	338
	Brazzaville	3	15	140	152	8	0	318
	Bulawayo	1	3	153	115	1	0	273
	Cotonou	3	0	6	64	126	0	199
	Eldoret	50	115	78	60	31	0	334
	Kampala	0	3	175	108	0	0	286
	Maputo	0	0	0	2	19	188	209
	Nairobi	0	8	144	132	0	0	284
	Windhoek	3	0	165	203	0	1	372
	**Total (% of 3246)**	**65 (2.0)**	**159 (4.9)**	**1299 (40.0)**	**1242 (38.3)**	**292 (9.0)**	**189 (5.8)**	**3246 (100)**

Supplementary Table 1B. Year of diagnosis and specific cancer types. Population-based cohort (n=3246). % as row-%.

		Patients per year of diagnosis					
		2010	2011	2012	2013	2014	2015
Cancer type	Breast	0.9%	2.3%	45.2%	38.1%	8.5%	5.0%
	Cervix	1.6%	3.7%	39.2%	39.4%	8.3%	7.9%
	Colorectum	3.0%	5.2%	39.4%	37.0%	12.0%	3.4%
	NHL	2.0%	8.5%	42.0%	34.3%	9.5%	3.7%
	Prostate	2.8%	6.3%	33.4%	41.9%	6.7%	8.9%
	**Total**	**2.0%**	**4.9%**	**40.0%**	**38.3%**	**9.0%**	**5.8%**

Supplementary Table 2. **Population-based cancer registries and study population characteristics**. Additional data sources: Population covered by population-based cancer registries, African Cancer Registry Network; Country human development index, World bank[7,51]

CRC, colorectal cancer; HDI, human development index; NHL, Non-Hodgkin lymphoma; PBCR, population-based cancer registry.

**General information on PBCR**				**Average patients registered in PBCR per year** (estimate based on registry data 2010-2015)					**Population-based sample** (n (% of patients eligible))					**Patients excluded** (n (% of population-based sample))					**Final population-based cohort** (n (% of population-based sample))					**Patients traced** (n (% of total cohort)				
**PBCR**	**Years observed**	**Popul-ation covered by PBCR**	**HDI 2015**	**Breast (2010-15)**	**Cervix (2010-15)**	**CRC (2010-15)**	**NHL (2011-14)**	**Prostate (2010-2015)**	**Breast**	**Cervix**	**CRC**	**NHL**	**Prostate**	**Breast**	**Cervix**	**CRC**	**NHL**	**Prostate**	**Breast**	**Cervix**	**CRC**	**NHL**	**Prostate**	**Breast**	**Cervix**	**CRC**	**NHL**	**Prostate**
**Abidjan Côte d'Invoire**	**2012-2013**	4.402.949	Low	265	232	53	56	143	80 (15)	80 (18)	80 (75)	59 (53)	80 (28)	17 (21)	13 (16)	12 (15)	16 (27)	8 (10)	63 (79)	67 (84)	68 (85)	43 (73)	72 (90)	50 (75.8)	54 (80.6)	54 (79.4)	30 (69.8)	57 (79)
**Addis Abeba Ethiopia**	**2012& 2014**	3.050.000	Low	437	228	138	51	25	137 (15)	114 (25)	85 (31)	86 (85)	18 (36)	23 (17)	68 (60)	3 (4)	18 (21)	8 (44)	114 (83)	46 (40)	82 (96)	68 (79)	10 (59)	51 (44.7)	46 (100)	37 (45.1)	33 (47.1)	10 (56)
**Bamako Mali**	**2012-2013**	1.810.366	Low	320	230	93	30	53	100 (15)	60 (13)	100 (54)	60 (98)	100 (95)	11 (11)	22 (37)	2(2)	8 (14)	39 (39)	89 (89)	38 (63)	98 (98)	52 (87)	61 (61)	48 (53.3)	38)100)	37 (37.8)	20 (37.8)	27 (28)
**Brazzaville Congo**	**2011-2014**	1.549.693	Low	106	164	25	11	116	79 (20)	80 (14)	58 (58)	42 (100)	80 (19)	4 (5)	0	0	3 (7)	14 (18)	75 (95)	80 (100)	58 (100)	39 (93)	66 (83)	19 (25.3)	21 (26.3)	13 (22.4)	6 (15.4)	27 (41)
**Bulawayo Zimbabwe**	**2012-2013**	653.000	Low	84	217	32	99	73	60 (35)	60 (14)	60 (96)	60 (30)	60 (41)	4 (7)	9 (15)	3 (5)	7 (12)	4 (6)	56 (93)	51 (85)	57 (95)	53 (88)	56 (93)	35 (62.5)	47 (91.2)	28 (49.1)	36 (67.9)	30 (53)

**Cotonou Benin**	**2013-2014**	678.874	Low	66	22	12	4	27	104 (78)	44 (100)	22 (100)	8 (100)	54 (100)	12 (12)	7 (16)	4 (18)	7 (88)	3 (6)	92 (88)	37 (84)	18 (82)	1 (13)	51 (94)	85 (92.4)	31 (83.9)	15 (83.3)	1 (100)	45 (88)
**Eldoret Kenya**	**2010-2014**	894.179	Medium	63	56	22	34	30	87 (69)	72 (39)	91 (100)	60 (78)	80 (67)	13 (15)	9 (13)	17 (19)	4 (8)	15 (19)	74 (85)	63 (88)	76 (84)	56 (93)	65 (81)	62 (76.5)	58 (92.1)	36 (47.4)	21 (36.8)	26 (35)
**Kampala Uganda**	**2011-2013**	2.010.000	Low	192	266	51	47	84	60 (16)	60 (12)	60 (59)	59 (63)	60 (35)	2 (3)	0	8 (13)	4 (7)	1 (2)	58 (97)	60 (100)	57 (95)	52 (88)	59 (98)	35 (60.3)	36 (60.0)	33 (57.9)	40 (72.7)	38 (64)
**Maputo Mozambique**	**2014-2015**	1.225.868	Low	54	95	18	18	71	45 (83)	124 (65)	25 (69)	25 (70)	66 (46)	2 (4)	70 (56.5)	1 (4)	1 (4)	2 (3)	43 (96)	54 (44)	24 (96)	24 (96)	64 (97)	34 (79.1)	54 (100)	13 (54.2)	17 (70.8)	28 (44)
**Nairobi Kenya**	**2012-2013**	3.138.369	Medium	356	248	123	98	205	60 (9)	60 (12)	60 (25)	60 (30)	60 (15)	3 (5)	2 (3)	2 (3,3)	7 (12)	2 (3)	57 (95)	58 (97)	58 (97)	53 (88)	58 (97)	37 (64.9)	46 (79.3)	40 (69.0)	44 (83.0)	45 (78)
**Namibia (nation-wide)**	**2012-2013**	2.104.900	Medium	227	237	95	80	223	80 (18)	80 (17)	80 (42)	80 (50)	80 (19)	3 (6)	4 (5)	3 (4)	14 (18)	2 (3)	75 (94)	76 (95)	77 (96)	66 (83)	78 (98)	61 (79.2)	59 (77.6)	57 (74.0)	45 (66.2)	32 (41)
**Subtotal**				2170	1995	644	528	1037	892 (41)	834 (41.2)	721 (112)	599 (113)	738 (71)	96 (11)	204 (25)	68 (9)	91 (15)	98 (13)	796 (89)	630 (76)	673 (93)	507 (85)	640 (87)	517 (63.9)	490 (77.8)	363 (53.9)	293 (56.8)	365 (53)
**Total:** 11 registries	2010-2015	21.518.198	8 Low 3 Medium	6374					3784 (29.6)					538 (14.2)					3246 (86)					2013 (62.0)				

Supplementary Table 2. **Population-based cancer registries and study population characteristics**. (contd.)

	**Follow-up below 30 days** (n (% of patients traced))				
**PBCR (years obs-erved)**	**Breast**	**Cervix**	**CRC**	**NHL**	**Prostate**
**Abidjan Côte d'Invoire**	14 (28.0)	18 (33:3)	13 (24.1)	11 (36.7)	10 (17.5)
**Addis Abeba Ethiopia**	5 (9.8)	6 (13.0)	6 (16.2)	3 (9.1)	1 (10.0)
**Bamako Mali**	6 (12.5)	6 (15.8)	2 (5.4)	5 (26.3)	5 (18.5)
**Brazzaville Congo**	1 (5.3)	0	1 (7.7)	0	4 (14.8)
**Bulawayo Zimbabwe**	8 (22.9)	16 (34.0)	6 (21.4)	16 (44.4)	10 (33.3)
**Cotonou Benin**	10 (11.8)	6 (19.4)	0	0	2 (4.4)
**Eldoret Kenya**	2 (3.6)	5 (8.6)	1 (2.8)	4 (19.0)	2 (7.7)
**Kampala Uganda**	12 (34.3)	14 (38.9)	8 (24.2)	15 (37.5)	11 (28.9)
**Maputo Mozambique**	0	2 (3.7)	4 (30.8)	3 (17.6)	2 (7.1)
**Nairobi Kenya**	1 (2.7)	4 (8.7)	2 (5.0)	4 (9.1)	9 (20.0)
**Namibia (nation-wide)**	1 (1.6)	2 (3.4)	5 (8.8)	2 (4.5)	1 (3.1)
**Subtotal**	60 (11.6)	79 (16.1)	48 (13.2)	63 (21.5)	57 (15.6)
**Total:** 11 registries	307 (15.3)				

Supplementary Figure 1. Map of Sub-Saharan Africa

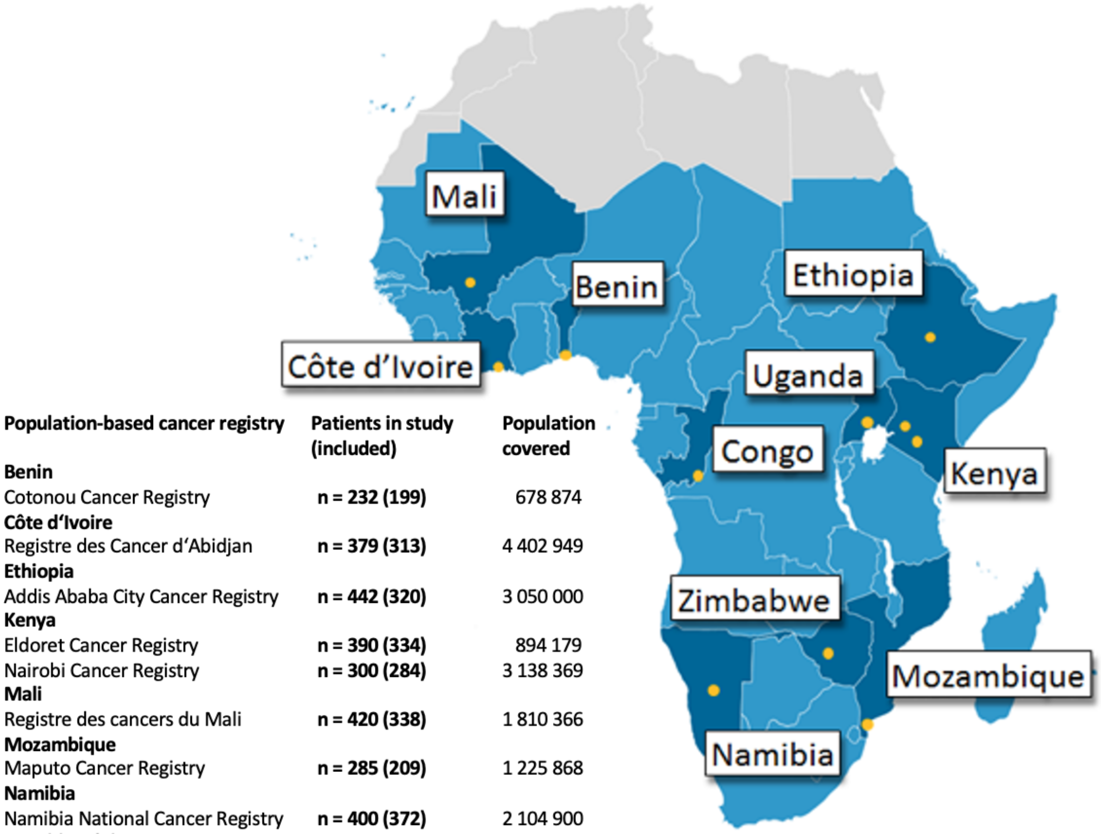

Population-based cancer registry	Patients in study (included)	Population covered
**Benin**		
Cotonou Cancer Registry	**n = 232 (199)**	678 874
**Côte d'Ivoire**		
Registre des Cancer d'Abidjan	**n = 379 (313)**	4 402 949
**Ethiopia**		
Addis Ababa City Cancer Registry	**n = 442 (320)**	3 050 000
**Kenya**		
Eldoret Cancer Registry	**n = 390 (334)**	894 179
Nairobi Cancer Registry	**n = 300 (284)**	3 138 369
**Mali**		
Registre des cancers du Mali	**n = 420 (338)**	1 810 366
**Mozambique**		
Maputo Cancer Registry	**n = 285 (209)**	1 225 868
**Namibia**		
Namibia National Cancer Registry	**n = 400 (372)**	2 104 900
**Republic of the Congo**		
Registre des cancers de Brazzaville	**n = 339 (318)**	1 549 693
**Uganda**		
Kampala Cancer Registry	**n = 299 (286)**	2 010 000
**Zimbabwe**		
Bulawayo Cancer Registry	**n = 300 (273)**	653 000

Supplementary Figure 1. **Map of Sub-Saharan Africa**. Countries and cities of participating population-based cancer registries are highlighted. On the left, the numbers included in the random sample are shown along with the covered population in the registry area. For details see also **Supplementary Table 2**.

Supplementary Figure 2. Flow chart

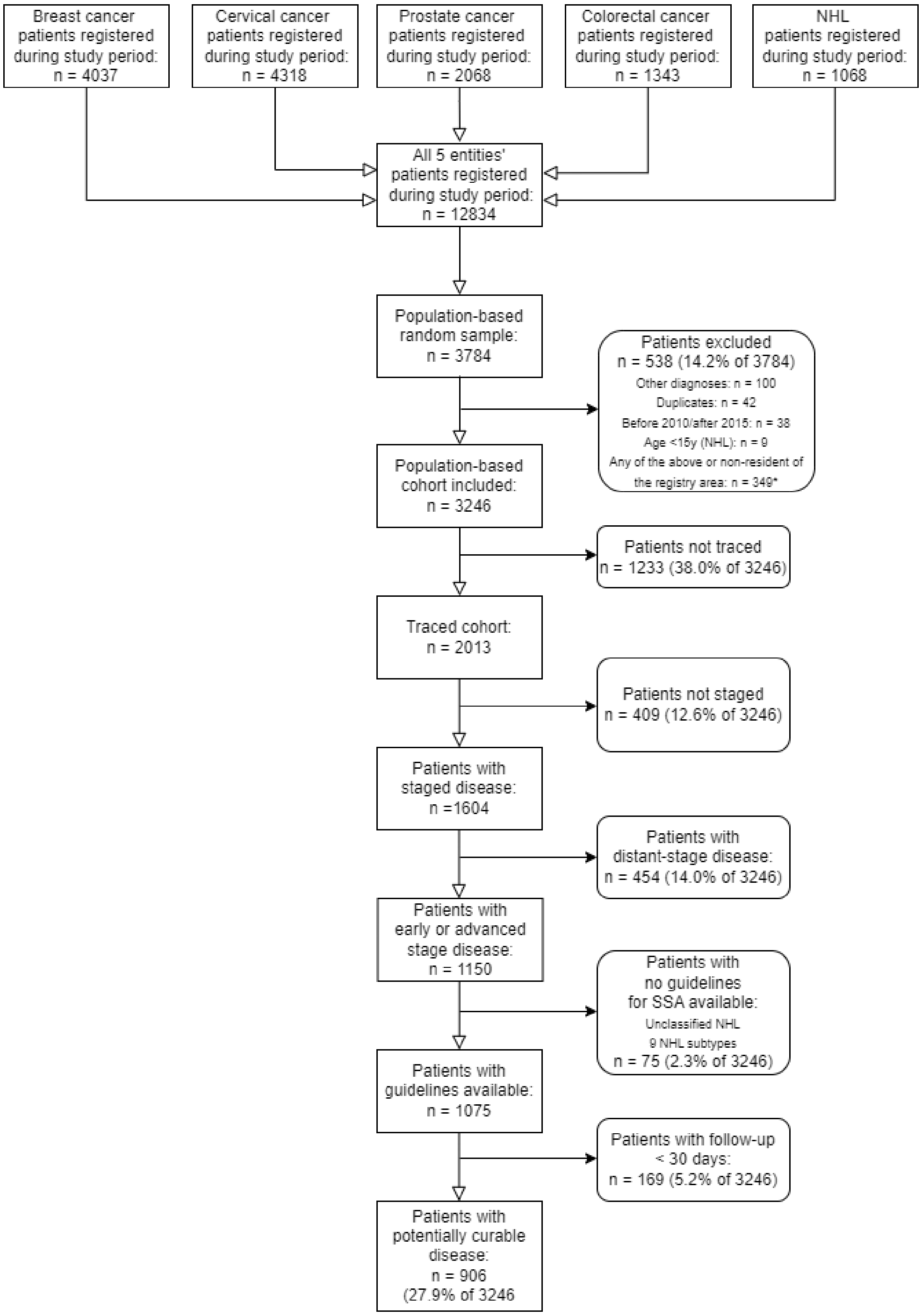

Supplementary Figure 2. **Detailed flowchart of patients**. Patients not traced: No information beyond cancer registry data available. *: detailed proportions were not available.

National Comprehensive Cancer Network; NHL, Non-Hodgkin lymphoma; NCCN, PF, prognostic factors; PC, prostate cancer; SSA, Sub-Saharan Africa.

Supplementary Table 3. Detailed therapy evaluation scheme

Supplementary Table 3. **Detailed comparative therapy evaluation scheme for guideline concordance[16,21–25] of potentially curable cancer** (see Excel file attached). Green: guideline concordant therapy; yellow: minor deviation from guideline concordant therapy; orange: major deviation from guideline concordant therapy. Red: No cancer-directed therapy or therapy without curable potential.

Supplementary Table 4. Traced patients by age, sex, cancer, registry

Supplementary Table 4. Traced patients in population-based cohort (n=3246). Stratified by age group, sex, cancer type and population-based cancer registry. % as row-%.

		n	Non-traced (%)	Traced (%)
	All	3246	38.0%	62.0%
**Age group** (years)	<30	232	45.7%	54.3%
	30-39	450	35.1%	64.9%
	40-49	605	32.2%	67.8%
	50-59	699	35.6%	64.4%
	60-69	601	40.4%	59.6%
	70-79	469	40.5%	59.5%
	>79	190	48.4%	51.6%
**Sex**	Female	1970	34.2%	65.8%
	Male	1276	43.9%	56.1%
**Cancer type**	Breast	796	36.4%	63.6%
	Prostate	640	43.0%	57.0%
	Cervix	630	22.2%	77.8%
	Colorectum	673	46.1%	53.9%
	NHL	507	43.0%	57.0%
	Abidjan	313	22.7%	77.3%

**Population-based cancer registry**	Addis	320	44.7%	55.3%
	Bamako	338	50.3%	49.7%
	Brazzaville	318	73.0%	27.0%
	Bulawayo	273	35.5%	64.5%
	Cotonou	199	11.6%	88.4%
	Eldoret	334	41.6%	58.4%
	Kampala	286	36.4%	63.6%
	Maputo	209	30.1%	69.9%
	Nairobi	284	25.4%	74.6%
	Windhoek	372	32.0%	68.0%

Supplementary Table 5. **Baseline characteristics and clinical diagnostics in population-based cohort** (n=3246), **stratified by cancer type**. % as column-%.

CT, computed tomography; ECOG PS, Eastern Cooperative Oncology Group Performance Status; FIGO, International Federation of Gynecology and Obstetrics; FNAC, fine needle aspiration cytology; HDI, human development index; MRI, magnetic resonance imaging; NHL, non-Hodgkin lymphoma; US, ultrasonography.

		**All n = 3246**	**Breast n = 796**	**Cervix n = 630**	**Prostate n = 640**	**Colorectum n = 673**	**NHL n = 507**
Population-based cancer registry	Abidjan, Côte d'Ivoire	313 (9.6)	63 (7.9)	67 (10.6)	72 (11.2)	68 (10.1)	43 (8.5)
	Addis Ababa, Ethiopia	320 (9.9)	114 (14.3)	46 (7.3)	10 (1.6)	82 (12.2)	68 (13.4)
	Bamako, Mali	338 (10.4)	89 (11.2)	38 (6.0)	61 (9.5)	98 (14.6)	52 (10.3)
	Brazzaville, Congo	318 (9.8)	75 (9.4)	80 (12.7)	66 (10.3)	58 (8.6)	39 (7.7)
	Bulawayo, Zimbabwe	273 (8.4)	56 (7.0)	51 (8.1)	56 (8.8)	57 (8.5)	53 (10.5)
	Cotonou, Benin	199 (6.1)	92 (11.6)	37 (5.9)	51 (8.0)	18 (2.7)	1 (0.2)
	Eldoret, Kenya	334 (10.3)	74 (9.3)	63 (10)	65 (10.2)	76 (11.3)	56 (11.0)
	Kampala, Uganda	286 (8.8)	58 (7.3)	60 (9.5)	59 (9.2)	57 (8.5)	52 (10.3)
	Maputo, Mozambique	209 (6.4)	43 (5.4)	54 (8.6)	64 (10.0)	24 (3.6)	24 (4.7)
	Nairobi, Kenya	284 (8.7)	57 (7.2)	58 (9.2)	58 (9.1)	58 (8.6)	53 (10.5)
	Namibia (nation-wide)	372 (11.5)	75 (9.4)	76 (12.1)	78 (12.2)	77 (11.4)	66 (13.0)
HDI	Low	2256 (69.5)	590 (74.1)	433 (68.7)	439 (68.6)	462 (68.6)	332 (65.5)
	Medium	990 (30.5)	206 (25.9)	197 (31.3)	201 (31.4)	211 (31.4)	175 (34.5)
Hospital type	Public	1674 (51.6)	396 (49.7)	395 (62.7)	335 (52.3)	299 (44.4)	249 (49.1)
	Private	419 (12.9)	119 (14.9)	46 (7.3)	95 (14.8)	103 (15.3)	56 (11)
	Unknown	1153 (35.5)	281 (35.3)	189 (30.0)	210 (32.8)	271 (40.3)	202 (39.8)
	No follow-up	569 (17.5)	124 (15.6)	57 (9.0)	115 (18.0)	146 (21.7)	127 (25)
	<30 days	447 (13.8)	81 (10.2)	90 (14.3)	100 (15.6)	91 (13.5)	85 (16.8)
Follow-Up	>=30; <90 days	300 (9.2)	71 (8.9)	68 (10.8)	48 (7.5)	61 (9.1)	52 (10.3)
	>=90 days	1930 (59.5)	520 (65.3)	415 (65.9)	377 (58.9)	375 (55.7)	243 (47.9)
Gender	Female	1970 (60.7)	796 (100)	630 (100)	n/a	324 (48.1)	220 (43.4)
	Male	1276 (39.3)	n/a	n/a	640 (100)	349 (51.9)	287 (56.6)
Age group (years)	15-29	232 (7.1)	48 (6.0)	26 (4.1)	0	63 (9.4)	95 (18.7)
	30-39	450 (13.9)	150 (18.8)	110 (17.5)	5 (0.8)	82 (12.2)	103 (20.3)
	40-49	605 (18.6)	226 (28.4)	154 (24.4)	4 (0.6)	123 (18.3)	98 (19.3)
	50-59	699 (21.5)	202 (25.4)	166 (26.3)	67 (10.5)	159 (23.6)	105 (20.7)
	60-69	601 (18.5)	108 (13.6)	95 (15.1)	209 (32.7)	137 (20.4)	52 (10.3)
	70-79	469 (14.4)	45 (5.7)	53 (8.4)	241 (37.7)	82 (12.2)	48 (9.5)
	80-89	167 (5.1)	16 (2)	24 (3.8)	99 (15.5)	24 (3.6)	4 (0.8)
	90-108	23 (0.7)	1 (0.1)	2 (0.3)	15 (2.3)	3 (0.4)	2 (0.4)
	Clinical	207 (6.4)	46 (5.8)	48 (7.6)	45 (7.0)	49 (7.3)	19 (3.7)

Base of diagnosis	Clinical incl USS, x -ray found	219 (6.7)	34 (4.3)	49 (7.8)	68 (10.6)	60 (8.9)	8 (1.6)
		906 (100)	277 (100)	263 (100)	100 (100)	189 (100)	77 (100)
	Biochemical, immunotest	56 (1.7)	-	-	55 (8.6)	-	1 (0.2)
	Surgery	80 (2.5)	39 (4.9)	1 (0.2)	32 (5)	8 (1.2)	-
	Cytology or haematology	358 (11)	204 (25.6)	10 (1.6)	1 (0.2)	60 (8.8)	83 (16.4)
	Histology of primary site	2223 (68.5)	431 (54.1)	522 (82.9)	428 (66.9)	486 (72.2)	356 (70.2)
	Histology of metastasis	19 (0.6)	6 (0.8)	0	3 (0.5)	6 (0.9)	4 (0.8)
	unknown	84 (2.6)	36 (4.5)	0	8 (1.2)	4 (0.6)	36 (7.1)
ECOG performance status	ECOG PS 0 or 1	460 (14.2)	144 (18.1)	119 (18.9)	67 (10.5)	79 (11.7)	51 (10.1)
	ECOG PS 2-4	425 (13.1)	54 (6.8)	100 (15.9)	94 (14.7)	96 (14.3)	81 (16.0)
	ECOG PS unknown	1128 (34.8)	308 (38.7)	271 (43)	204 (31.9)	188 (27.9)	157 (31.0)
	Medical record not found	1233 (38)	290 (36.4)	140 (22.2)	275 (43.0)	310 (46.1)	218 (43.0)
HIV status	Negative	308 (9.5)	65 (8.2)	81 (12.9)	50 (7.8)	55 (8.2)	57 (11.2)
	Positive	239 (7.4)	29 (3.6)	96 (15.2)	4 (0.6)	14 (2.1)	96 (18.9)
	No HIV status identified	1466 (45.2)	412 (51.8)	313 (49.7)	311 (48.6)	294 (43.7)	136 (26.8)
	Medical record not found	1233 (38)	290 (36.4)	140 (22.2)	275 (43)	310 (46.1)	218 (43)
Stage	**Early** (TNM I/II, FIGO I-IIa, Ann Arbor I/II (aggressive NHL histopathological subtypes and unclassified NHL)	387 (11.9)	124 (15.6)	87 (13.8)	44 (8.7)	54 (8.0)	48 (9.5)
	**Advanced** (TNM III (BC and PC), TNM I/II (high risk CRC) and TNM III (CRC), FIGO IIb-IIIb, Ann Arbor III/IV (high-grade NHL histopathological subtypes and unclassified NHL))	763 (23.5)	187 (23.5)	222 (35.2)	112 (22.1)	159 (23.6)	112 (22.1)
	**Distant** TNM IV, FIGO IV, all low-grade NHL (histopathologically sub-classified, any stage)	454 (14)	85 (10.7)	117 (18.6)	61 (12.0)	96 (14.3)	20 (3.9)
	Not staged (excl. low-grade NHL)	409 (12.6)	110 (13.8)	64 (10.2)	94 (18.5)	54 (8.0)	109 (21.5)
	Medical record not found (excl. low-grade NHL)	1233 (38)	290 (36.4)	140 (22.2)	196 (38.6)	310 (46.1)	218 (43.0)
Imaging	US/X-Ray	495 (15.2)	206 (25.9)	127 (20.2)	151 (23.6)	84 (12.5)	78 (15.4)
	CT/MRT	234 (7.2)	29 (3.6)	55 (8.7)	69 (10.8)	114 (16.9)	36 (7.1)
	no imaging documented	1284 (39.6)	271 (34)	308 (48.9)	145 (22.7)	165 (24.5)	175 (34.5)
	Medical record not found	1233 (38)	290 (36.4)	140 (22.2)	275 (43.0)	310 (46.1)	218 (43.0)

Supplementary Table 6. Treatment by cancer type: n=3246

Supplementary Table 6. **Overall Treatment in population-based cohort** (n=3246), **stratified by cancer type**. % as column-%. In 16 of 1233 non-traced patients, some information on treatment was documented in cancer registries. For the remaining 1217, no information on treatment was registered.

BT, brachytherapy; CDT, cancer treatment; EBRT, external beam radiation therapy; NHL, Non-Hodgkin lymphoma; PBCR, population-based cancer registry; RT, radiotherapy; Sx, surgery.

	**All**<br>**n = 3246**	**Breast**<br>**n = 796**	**Cervix**<br>**n = 630**	**Prostate**<br>**n = 640**	**Colorectal**<br>**n = 673**	**NHL**<br>**n = 507**
Any cancer-directed therapy	1446 (44.5)	407 (51.1)	275 (43.7)	271 (42.3)	291 (43.2)	202 (39.8)
None/not applicable	583 (18.0)	99 (12.4)	215 (34.1)	94 (14.7)	72 (10.7)	103 (20.3)
Not traced, no info in PBCR	1217 (37.5)	290 (36.4)	140 (22.2)	275 (43.0)	310 (46.1)	202 (39.8)
Any chemotherapy	801 (24.7)	281 (35.3)	129 (20.5)	13 (2.0)	195 (29)	183 (36.1)
None/not applicable	1228 (37.8)	225 (28.3)	361 (57.3)	352 (55.0)	168 (25)	122 (24.1)
Not traced, no info in PBCR	1217 (37.5)	290 (36.4)	140 (22.2)	275 (43.0)	310 (46.1)	202 (39.8)
Any hormonal therapy	328 (22.8)	142 (17.8)	n/a	186 (29.1)	n/a	n/a
None/not applicable	543 (37.8)	364 (45.7)	n/a	179 (28.0)	n/a	n/a
Not traced, no info in PBCR	565 (39.3)	290 (36.4)	n/a	275 (43.0)	n/a	n/a
Any radiotherapy (EBRT+/-BT)	435 (13.4)	127 (16)	141 (22.4)	73 (11.4)	64 (9.5)	30 (5.9)
None/not applicable	1594 (49.1)	379 (47.6)	349 (55.4)	292 (45.6)	299 (44.4)	275 (54.2)
Not traced, no info in PBCR	1217 (37.5)	290 (36.4)	140 (22.2)	275 (43)	310 (46.1)	202 (39.8)
Any cancer-directed surgery	786 (24.2)	324 (40.7)	92 (14.6)	108 (16.9)	237 (35.2)	25 (4.9)
None/not applicable	1243 (38.3)	182 (22.9)	398 (63.2)	257 (40.2)	126 (18.7)	280 (55.2)
Not traced, no info in PBCR	1217 (37.5)	290 (36.4)	140 (22.2)	275 (43.0)	310 (46.1)	202 (39.8)

Supplementary Figure 3. Treatment categories of patients in population-based cohort (n=3246)

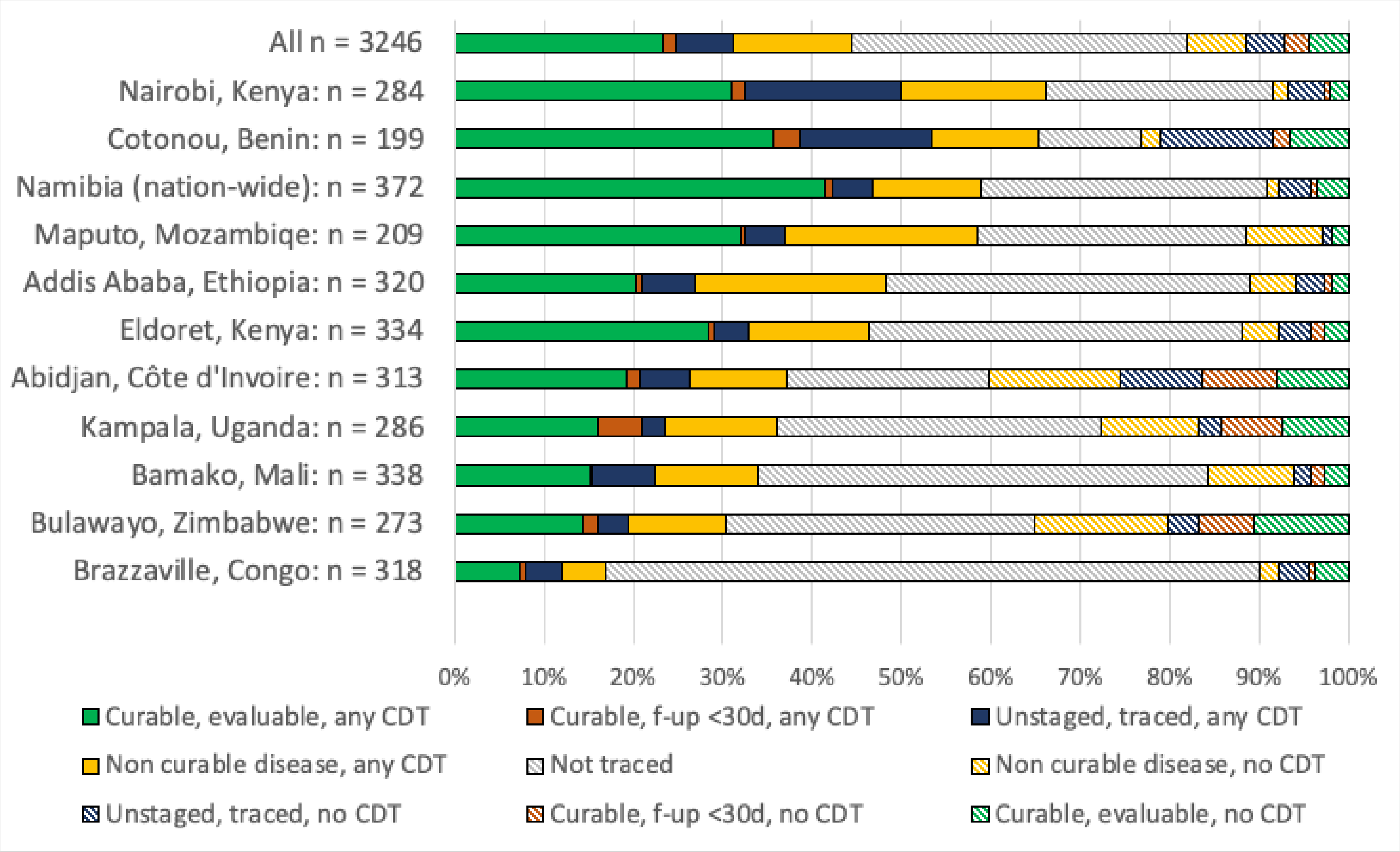

Supplementary Figure 3. **Treatment categories of patients adjusted for curability of disease, follow-up, staging and tracing in population-based cohort** (n=3246), **stratified by cancer registry**. Bars filled with green, brown, blue and yellow: any cancer treatment (CDT); striped bars with corresponding colors: no cancer treatment; grey striped bar: not traced. Curable (green and brown) refers to stage I-III breast, cervix, colorectal and prostate cancer and aggressive Non-Hodgin lymphoma histopathological subtype, any stage. Non-curable (yellow) refers to breast, cervix, colorectal and prostate cancer stage IV and indolent/unclassified Non-Hodgkin lymphoma.

CDT, cancer treatment; f-up, follow-up; 30d, 30 days

Supplementary Table 7. Details on overall treatment and guideline concordance by cancer type

Supplementary Table 7. **Details on overall treatment and guideline concordance**[16,21–25] **in patients with potentially curable cancer** (n=906), **stratified by cancer type**. % as column-%. Evaluation of guideline concordance refers to Table 1 and Supplementary Table 1.

BT, brachytherapy; CDT, cancer treatment; EBRT, external beam radiation therapy; NHL, Non-Hodgkin lymphoma; RT, radiotherapy; Sx, surgery.

	**All n=906**	**Breast n=277**	**Cervix n=263**	**Prostate n=100**	**Colorectal n=189**	**NHL n=77**
Proportion of potentially curable cancer (%) among population-based cohort	27.9	34.9	41.2	15.6	28.1	15.2
Any cancer-directed therapy	759 (83.8)	251 (90.6)	195 (74.1)	81 (81.0)	172 (91.0)	60 (77.9)
None	147 (16.2)	26 (9.4)	68 (25.9)	19 (19.0)	17 (9.0)	17 (22.1)
Any chemo(-immuno-)therapy	459 (50.7)	185 (66.8)	91 (34.6)	3 (3.0)	124 (65.6)	56 (72.7)
None	447 (49.3)	92 (33.2)	172 (65.4)	97 (97.0)	65 (34.4)	21 (27.3)
Any hormonal therapy	154 (40.8)	99 (35.7)	n/a	55 (55.0)	n/a	n/a
None	223 (59.2)	178 (64.3)	n/a	45 (45.0)	n/a	n/a
Any radiotherapy (EBRT+/-BT)	268 (29.6)	87 (31.4)	104 (39.5)	20 (20)	37 (19.6)	20 (26.0)
None	638 (70.4)	190 (68.6)	159 (60.5)	80 (80.0)	152 (80.4)	57 (74.0)
Any cancer-directed surgery	480 (53.0)	211 (76.2)	68 (25.9)	40 (40.0)	155 (82)	6 (7.8)
None	426 (47.0)	66 (23.8)	195 (74.1)	60 (60.0)	34 (18.0)	71 (92.2)
Guideline concordant	104 (11.5)	n/a	48 (18.3)	38 (38.0)	8 (4.2)	10 (13.0)
Guideline concordant **or** minor deviation	151 (16.7)	151 (54.5)	n/a	n/a	n/a	n/a
Minor deviation	118 (13.0)	n/a	25 (9.5)	1 (1.0)	56 (29.6)	36 (46.8)
Major deviation	273 (30.1)	100 (36.1)	54 (20.5)	34 (34.0)	75 (39.7)	10 (13)
No CDT or therapy without curable potential	260 (28.7)	26 (9.4)	136 (51.7)	27 (27.0)	50 (26.5)	21 (27.3)

Supplementary Table 8. Treatment by registry: n=3246

Supplementary Table 8. **Treatment by type in population-based cohort** (n=3246), **stratified by registry**. % as column-%. In 16 of 1233 non-traced patients, some information on treatment was documented in cancer registries. For the remaining 1217, no information on treatment was registered.

BT, brachytherapy; CDT, cancer treatment; EBRT, external beam radiation therapy; NHL, Non-Hodgkin lymphoma; PBCR, population-based cancer registry; RT, radiotherapy; Sx, surgery.

	All n = 3246	Abidjan Côte d'Ivoire n = 313	Addis Ababa Ethiopia n = 320	Bamako Mali n = 338	Brazzaville Congo n = 318	Bulawayo Zimbabwe n = 273	Cotonou Benin n = 199	Eldoret Kenya n = 334	Kampala Uganda n = 286	Maputo Mozambique n = 209	Nairobi Kenya n = 284	Namibia (nation-wide) n = 372
Breast	796 (24.5)	63 (20.1)	114 (35.6)	89 (26.3)	75 (23.6)	56 (20.5)	92 (46.2)	74 (22.2)	58 (20.3)	43 (20.6)	57 (20.1)	75 (20.2)
Cervix	630 (19.4)	67 (21.4)	46 (14.4)	38 (11.2)	80 (25.2)	51 (18.7)	37 (18.6)	63 (18.9)	60 (21)	54 (25.8)	58 (20.4)	76 (20.4)
Colorectal	673 (20.7)	68 (21.7)	82 (25.6)	98 (29)	58 (18.2)	57 (20.9)	18 (9)	76 (22.8)	57 (19.9)	24 (11.5)	58 (20.4)	77 (20.7)
NHL	507 (15.6)	43 (13.7)	68 (21.2)	52 (15.4)	39 (12.3)	53 (19.4)	1 (0.5)	56 (16.8)	52 (18.2)	24 (11.5)	53 (18.7)	66 (17.7)
Prostate	640 (19.7)	72 (23)	10 (3.1)	61 (18)	66 (20.8)	56 (20.5)	51 (25.6)	65 (19.5)	59 (20.6)	64 (30.6)	58 (20.4)	78 (21)
Any cancer-directed therapy	1446 (44.5)	118 (37.7)	156 (48.8)	117 (34.6)	54 (17)	84 (30.8)	130 (65.3)	155 (46.4)	101 (35.3)	122 (58.4)	189 (66.5)	220 (59.1)
None	583 (18)	124 (39.6)	34 (10.6)	51 (15.1)	32 (10.1)	95 (34.8)	46 (23.1)	40 (12)	81 (28.3)	24 (11.5)	23 (8.1)	33 (8.9)
Not traced, no info in PBCR	1217 (37.5)	71 (22.7)	130 (40.6)	170 (50.3)	232 (73)	94 (34.4)	23 (11.6)	139 (41.6)	104 (36.4)	63 (30.1)	72 (25.4)	119 (32)
Any chemotherapy	801 (24.7)	46 (14.7)	100 (31.2)	85 (25.1)	24 (7.5)	38 (13.9)	34 (17.1)	114 (34.1)	27 (9.4)	97 (46.4)	112 (39.4)	124 (33.3)
None/not applicable	1228 (37.8)	196 (62.6)	90 (28.1)	83 (24.6)	62 (19.5)	141 (51.6)	142 (71.4)	81 (24.3)	155 (54.2)	49 (23.4)	100 (35.2)	129 (34.7)
Not traced, no info in PBCR	1217 (37.5)	71 (22.7)	130 (40.6)	170 (50.3)	232 (73)	94 (34.4)	23 (11.6)	139 (41.6)	104 (36.4)	63 (30.1)	72 (25.4)	119 (32)
Any hormonal therapy	328 (10.1)	37 (11.8)	31 (9.7)	24 (7.1)	15 (4.7)	9 (3.3)	36 (18.1)	35 (10.5)	15 (5.2)	33 (15.8)	33 (11.6)	60 (16.1)
None/not applicable	1701 (52.4)	205 (65.5)	159 (49.7)	144 (42.6)	71 (22.3)	170 (62.3)	140 (70.4)	160 (47.9)	167 (58.4)	113 (54.1)	179 (63)	193 (51.9)
Not traced, no info in PBCR	1217 (37.5)	71 (22.7)	130 (40.6)	170 (50.3)	232 (73)	94 (34.4)	23 (11.6)	139 (41.6)	104 (36.4)	63 (30.1)	72 (25.4)	119 (32)
Any radiotherapy (EBRT+/-BT)	435 (13.4)	8 (2.6)	54 (16.9)	9 (2.7)	8 (2.5)	9 (3.3)	7 (3.5)	32 (9.6)	56 (19.6)	8 (3.8)	100 (35.2)	144 (38.7)
None/not applicable	1594 (49.1)	234 (74.8)	136 (42.5)	159 (47)	78 (24.5)	170 (62.3)	169 (84.9)	163 (48.8)	126 (44.1)	138 (66)	112 (39.4)	109 (29.3)
Not traced, no info in PBCR	1217 (37.5)	71 (22.7)	130 (40.6)	170 (50.3)	232 (73)	94 (34.4)	23 (11.6)	139 (41.6)	104 (36.4)	63 (30.1)	72 (25.4)	119 (32)
Any cancer-directed surgery	786 (24.2)	59 (18.8)	91 (28.4)	94 (27.8)	31 (9.7)	54 (19.8)	98 (49.2)	93 (27.8)	33 (11.5)	50 (23.9)	80 (28.2)	103 (27.7)
None/not applicable	1243 (38.3)	183 (58.5)	99 (30.9)	74 (21.9)	55 (17.3)	125 (45.8)	78 (39.2)	102 (30.5)	149 (52.1)	96 (45.9)	132 (46.5)	150 (40.3)
Not traced, no info in PBCR	1217 (37.5)	71 (22.7)	130 (40.6)	170 (50.3)	232 (73)	94 (34.4)	23 (11.6)	139 (41.6)	104 (36.4)	63 (30.1)	72 (25.4)	119 (32)

Supplementary Table 9. Overall treatment and guideline concordance by registry

Supplementary Table 9. Overall treatment and guideline concordance[16,21–25] in patients with potentially curable cancer (n=906), **stratified by registry**. % as column-%. Evaluation of guideline concordance refers to Table 1 and Supplementary Table 1.

BT, brachytherapy; CDT, cancer treatment; EBRT, external beam radiation therapy; NHL, Non-Hodgkin lymphoma; PBCR, population-based cancer registry; RT, radiotherapy; Sx, surgery

	**All** **n=906** (27.9% of 3246)	**Abidjan Côte d'Ivoire** **n=85** (27.2% of 313)	**Addis Ababa Ethiopia** **n=71** (22.2% of 320)	**Bamako Mali** **n=60** (17.7% of 338)	**Brazzaville Congo** **n=35** (11.0% of 318)	**Bulawayo Zimbabwe** **n=68** (24.9% of 273)	**Cotonou Benin** **n=84** (42.2% of 199)	**Eldoret Kenya** **n=104** (31.1% of 334)	**Kampala Uganda** **n=67** (23.4% of 286)	**Maputo Mozambique** **n=71** (34.0% of 209)	**Nairobi Kenya** **n=94** (33.1% of 284)	**Namibia nation-wide** **n=167** (44.9% of 372)
Breast	277 (30.6)	21 (24.7)	20 (28.2)	23 (38.3)	10 (28.6)	18 (26.5)	44 (52.4)	31 (29.8)	17 (25.4)	24 (33.8)	23 (24.5)	46 (27.5)
Cervix	263 (29)	15 (17.6)	26 (36.6)	12 (20)	6 (17.1)	27 (39.7)	11 (13.1)	46 (44.2)	21 (31.3)	28 (39.4)	32 (34)	39 (23.4)
Colorectum	189 (20.9)	27 (31.8)	16 (22.5)	22 (36.7)	8 (22.9)	14 (20.6)	11 (13.1)	17 (16.3)	19 (28.4)	8 (11.3)	14 (14.9)	33 (19.8)
NHL	77 (8.5)	4 (4.7)	8 (11.3)	2 (3.3)	1 (2.9)	2 (2.9)	0	0	5 (7.5)	1 (1.4)	18 (19.1)	36 (21.6)
Prostate	100 (11)	18 (21.2)	1 (1.4)	1 (1.7)	10 (28.6)	7 (10.3)	18 (21.4)	10 (9.6)	5 (7.5)	10 (14.1)	7 (7.4)	13 (7.8)
Any cancer-directed treatment	759 (83.8)	60 (70.6)	65 (91.5)	51 (85)	23 (65.7)	39 (57.4)	71 (84.5)	95 (91.3)	46 (68.7)	67 (94.4)	88 (93.6)	154 (92.2)
None	147 (16.2)	25 (29.4)	6 (8.5)	9 (15)	12 (34.3)	29 (42.6)	13 (15.5)	9 (8.7)	21 (31.3)	4 (5.6)	6 (6.4)	13 (7.8)
Any chemotherapy	459 (50.7)	26 (30.6)	41 (57.7)	42 (70)	11 (31.4)	19 (27.9)	21 (25)	70 (67.3)	8 (11.9)	57 (80.3)	61 (64.9)	103 (61.7)
None/not applicable	447 (49.3)	59 (69.4)	30 (42.3)	18 (30)	24 (68.6)	49 (72.1)	63 (75)	34 (32.7)	59 (88.1)	14 (19.7)	33 (35.1)	64 (38.3)
Any hormonal therapy	154 (17)	16 (18.8)	14 (19.7)	6 (10)	5 (14.3)	5 (7.4)	17 (20.2)	20 (19.2)	4 (6)	16 (22.5)	14 (14.9)	37 (22.2)
None/not applicable	752 (83)	69 (81.2)	57 (80.3)	54 (90)	30 (85.7)	63 (92.6)	67 (79.8)	84 (80.8)	63 (94)	55 (77.5)	80 (85.1)	130 (77.8)
Any radiotherapy (EBRT+/-BT)	268 (29.6)	6 (7.1)	29 (40.8)	3 (5)	5 (14.3)	6 (8.8)	6 (7.1)	25 (24)	32 (47.8)	5 (7)	52 (55.3)	99 (59.3)
None/not applicable	638 (70.4)	79 (92.9)	42 (59.2)	57 (95)	30 (85.7)	62 (91.2)	78 (92.9)	79 (76)	35 (52.2)	66 (93)	42 (44.7)	68 (40.7)
Any cancer-directed surgery	480 (53)	39 (45.9)	45 (63.4)	43 (71.7)	16 (45.7)	31 (45.6)	62 (73.8)	64 (61.5)	19 (28.4)	33 (46.5)	46 (48.9)	82 (49.1)

None/not applicable	426 (47)	46 (54.1)	26 (36.6)	17 (28.3)	19 (54.3)	37 (54.4)	22 (26.2)	40 (38.5)	48 (71.6)	38 (53.5)	48 (51.1)	85 (50.9)
Guideline concordant	151 (16.7)	4 (4.7)	20 (28.2)	14 (23.3)	2 (5.7)	8 (11.8)	7 (8.3)	22 (21.2)	3 (4.5)	19 (26.8)	16 (17)	36 (21.6)
Guideline concordant **or** minor deviation	104 (11.5)	10 (11.8)	4 (5.6)	4 (6.7)	5 (14.3)	3 (4.4)	6 (7.1)	20 (19.2)	3 (4.5)	4 (5.6)	15 (16)	30 (18)
Minor deviation	118 (13)	8 (9.4)	11 (15.5)	6 (10)	2 (5.7)	6 (8.8)	4 (4.8)	6 (5.8)	3 (4.5)	1 (1.4)	15 (16)	56 (33.5)
Major deviation	273 (30.1)	33 (38.8)	18 (25.4)	20 (33.3)	13 (37.1)	14 (20.6)	44 (52.4)	19 (18.3)	31 (46.3)	20 (28.2)	32 (34)	29 (17.4)
No CDT or therapy without curable potential	260 (28.7)	30 (35.3)	18 (25.4)	16 (26.7)	13 (37.1)	37 (54.4)	23 (27.4)	37 (35.6)	27 (40.3)	27 (38)	16 (17)	16 (9.6)

Supplementary Table 10. Proportion of GCT by cancer type and registry in PCD (n=906)

Supplementary Table 10. **Proportion of guideline-concordant treatment** (GCT) **by cancer type and registry in patients with potentially curable cancer** (PCD, n=906).

GCT, guideline-concordant treatment or minor deviation; Non-GCT, major deviation from guidelines or no cancer-directed therapy or therapy without curative potential.

		Breast				Prostate				Cervix				Colorectum				NHL (=DLBCL/Burkitt lymphoma)			
		GCT		Non-GCT		GCT		Non-GCT		GCT		Non-GCT		GCT		Non-GCT		GCT		Non-GCT	
		n	%	n	%	n	%	n	%	n	%	n	%	n	%	n	%	n	%	n	%
Registry	Abidjan	4	19,05%	17	80,95%	2	11,11%	16	88,89%	5	33,33%	10	66,67%	7	25,93%	20	74,07%	4	100%	0	0
	Addis	20	100,00%	0	0,00%	0	0,00%	1	100,00%	4	15,38%	22	84,62%	5	31,25%	11	68,75%	6	75,00%	2	25,00%
	Bamako	14	60,87%	9	39,13%	0	0,00%	1	100,00%	3	25,00%	9	75,00%	7	31,82%	15	68,18%	0	0%	2	100%
	Brazzaville	2	20,00%	8	80,00%	4	40,00%	6	60,00%	1	16,67%	5	83,33%	2	25,00%	6	75,00%	0	0%	1	100%
	Bulawayo	8	44,44%	10	55,56%	2	28,57%	5	71,43%	1	3,70%	26	96,30%	6	42,86%	8	57,14%	0	0%	2	100%
	Cotonou	7	15,91%	37	84,09%	3	16,67%	15	83,33%	2	18,18%	9	81,82%	5	45,45%	6	54,55%	0	0%	0	100%
	Eldoret	22	70,97%	9	29,03%	4	40,00%	6	60,00%	16	34,78%	30	65,22%	6	35,29%	11	64,71%	0	0%	0	100%
	Kampala	3	17,65%	14	82,35%	3	60,00%	2	40,00%	1	4,76%	20	95,24%	1	5,26%	18	94,74%	1	20,00%	4	80,00%
	Maputo	19	79,17%	5	20,83%	3	30,00%	7	70,00%	1	3,57%	27	96,43%	1	12,50%	7	87,50%	0	0%	1	100%
	Nairobi	16	69,57%	7	30,43%	5	71,43%	2	28,57%	8	25,00%	24	75,00%	5	35,71%	9	64,29%	12	66,60%	6	33,40%
	Windhoek	36	78,26%	10	21,74%	13	100,00%	0	0,00%	31	79,49%	8	20,51%	19	57,58%	14	42,42%	23	47,60%	13	22,00%
	Subtotal per cancer type	151		126		39		61		73		190		64		125		46		31	
	Total per cancer type	277				100				263				189				77			

Supplementary Figure 4. Supplementary multivariable Poisson regression in PCD (n=906), adjusted by registry

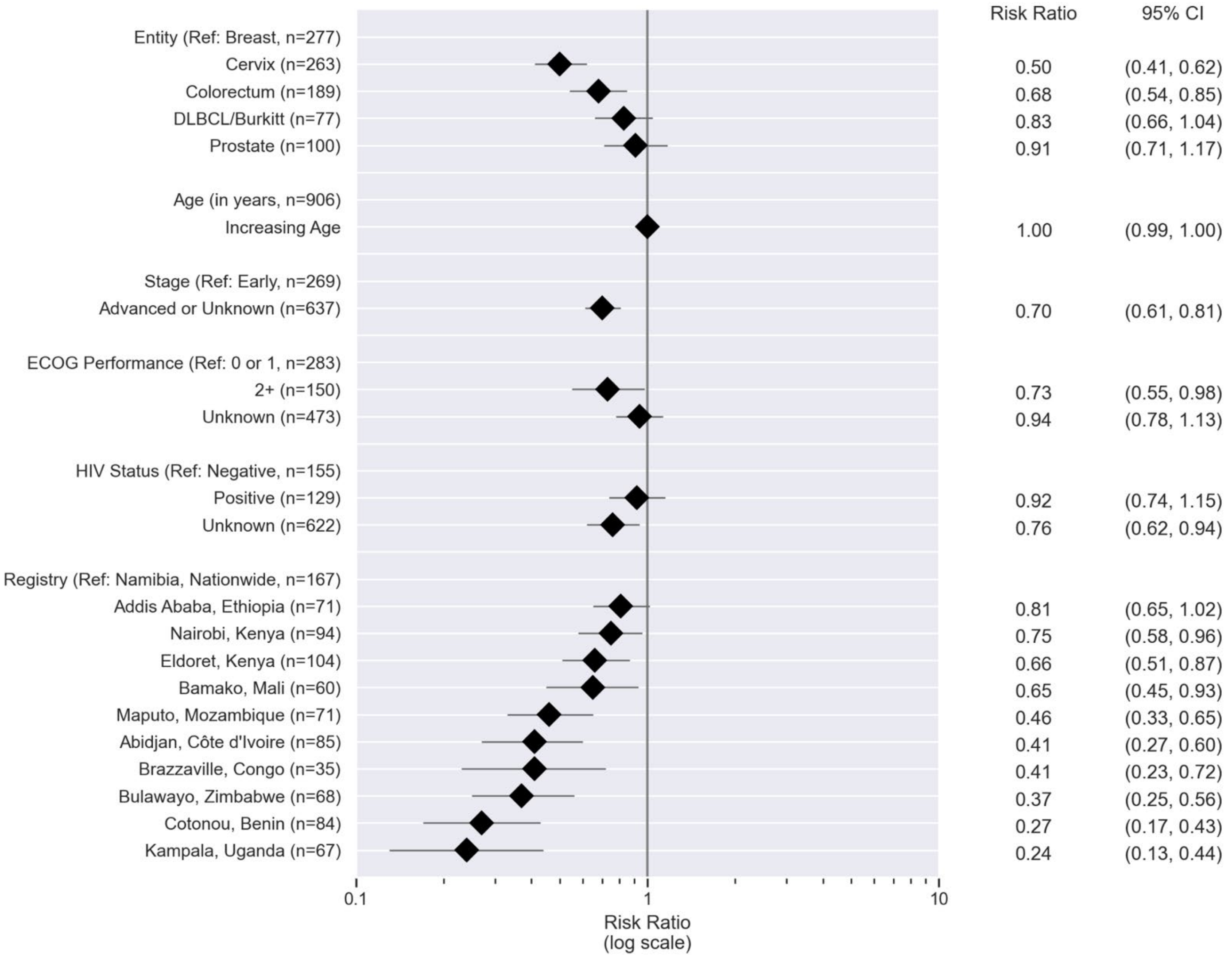

Supplementary Figure 4. Factors associated with guideline concordant therapy treatment (GCT) in patients with potentially curable cancer (n=906, multivariable modified Poisson regression; adjusted by registry)**.** GCT refers to combined guideline concordance or minor deviation from guidelines[16,21–25]. Evaluation of guideline concordance refers to Table 1 and Supplementary Table 1. Note that at the time of the study, functioning radiotherapy facilities were in place only in Namibia, Addis Ababa, Nairobi and Abidjan.

DLBCL, diffuse large B-cell lymphoma; ECOG, Eastern Cooperative Oncology Group; Maj. Dev., major deviation; No Tx, no treatment or treatment without curative potential
